# Midterm Clinical and Radiographic Outcomes of Transarticular Trephonic Plasty-Assisted Percutaneous Endoscopic Lumbar Interbody Fusion for Single-Level Lumbar Degenerative Disease: A Retrospective Cohort Study

**DOI:** 10.3390/jcm15207980

**Published:** 2026-10-15

**Authors:** Xiaohao Sun, Junliang Xu, Yukai Cui, Shiju Yang, Xilong Cui, Yunpeng Wan, Haiyang Yu, Wen Yin, Wei Jiao

**Affiliations:** 1 Department of Orthopaedics, Affiliated Fuyang People’s Hospital of Anhui Medical University, 501 Sanqing Road, Fuyang 236000, China; 2445012513@stu.ahmu.edu.cn (X.S.); 13665655468@163.com (Y.C.); 2645012717@stu.ahmu.edu.cn (S.Y.); cuixilong.wang@163.com (X.C.); wyp666202609@163.com (Y.W.); fy.yhy@163.com (H.Y.); 2 Department of Orthopaedics, Affiliated Fuyang People’s Hospital of Bengbu Medical University, 501 Sanqing Road, Fuyang 236000, China; 20251051387@stu.bbmu.edu.cn; 3 Clinical Research Center for Spinal Deformity of Anhui Province, Fuyang People’s Hospital, 501 Sanqing Road, Fuyang 236000, China

**Keywords:** lumbar degenerative disease, percutaneous endoscopic lumbar interbody fusion, transarticular trephonic plasty, Kambin’s triangle, endoscopic spine surgery, midterm outcomes

## Abstract

**Background/Objectives:** To evaluate midterm clinical, radiographic, and safety outcomes after transarticular trephonic plasty (TTP)-assisted percutaneous endoscopic lumbar interbody fusion (PE-LIF) for lumbar degenerative disease. **Methods:** This retrospective single-center cohort included 130 patients with complete clinical and imaging assessments and at least 24 months of follow-up. Clinical outcomes included back and leg pain visual analog scales, Oswestry Disability Index (ODI), Japanese Orthopaedic Association score, 36-Item Short Form Health Survey (SF-36) component scores, and modified MacNab outcomes. Radiographic measurements and computed tomography-based Bridwell fusion grades were assessed. Linear mixed-effects models included categorical visits, a patient-specific random intercept, and adjustment for final follow-up duration; multiple comparisons used Holm correction. **Results:** Median follow-up was 42.5 months (interquartile range, 36.0–50.75; range, 29–60). Mean operative time was 133.45 ± 15.63 min. Estimated blood loss derived from preoperative-to-postoperative-day-1 hematocrit change was 323.76 ± 66.24 mL. The estimated final-versus-preoperative ODI change was −40.15 points (95% confidence interval, −41.02 to −39.29). Clinical scores and radiographic measurements differed from baseline at all postoperative visits (all adjusted *p* < 0.05). Pain scores decreased from the early assessment to 6 months; functional and quality-of-life scores changed through 12 months, with a further increase in the SF-36 physical component score at final follow-up. Bridwell grade I or II fusion occurred in 118 patients (90.8%), and 119 (91.5%) had excellent or good MacNab outcomes. Complications occurred in 10 patients (7.7%); 2 required reoperation (1.5%). **Conclusions:** The analyzed cohort had sustained clinical improvement after TTP-assisted PE-LIF, with fusion, complication, and reoperation rates of 90.8%, 7.7%, and 1.5%, respectively. Controlled studies are needed to determine the contribution of TTP to these outcomes.

## 1. Introduction

Lumbar degenerative disease (LDD) is a common cause of low back and leg pain and functional impairment in middle-aged and older adults, substantially compromising their quality of life [1]. Occupational studies also document a substantial burden: Bryndal et al. reported low back pain in 85% of surveyed Polish paramedics, together with pain-related functional impairment [2]. For patients who do not respond to standardized conservative treatment and whose clinical symptoms are concordant with imaging findings, surgery is an important treatment option [3,4]. Traditional open fusion can achieve adequate decompression and reliable fusion but entails greater tissue trauma and a greater postoperative recovery burden. Minimally invasive transforaminal lumbar interbody fusion (MIS-TLIF) reduces soft-tissue injury but does not eliminate disruption of the paraspinal tissues [5,6].

With advances in spinal endoscopic techniques, percutaneous endoscopic lumbar interbody fusion (PE-LIF) combines the advantages of endoscopic decompression and interbody fusion, including reduced tissue injury, faster postoperative recovery, and less iatrogenic damage [7,8,9]. However, the restricted operative space inherent in uniportal endoscopy remains a technical limitation. Establishing working access through the transarticular approach is therefore a key determinant of operative efficiency and procedural continuity in PE-LIF. Conventional techniques require repeated soft-tissue exposure, hemostasis, and incremental bone resection, which may prolong operative time and compromise endoscopic visualization.

A computed tomography (CT)-based anatomical study showed that the posterolateral projection of the center of Kambin’s triangle corresponds to the superolateral quadrant of the articular process, which provides an anatomical localization target for the transarticular approach [10]. Under fluoroscopic guidance, transarticular trephonic plasty (TTP) involves anchoring a Kirschner wire (K-wire) in this region, advancing a coaxial trephine over the K-wire, and resecting the articular process en bloc to create an osseous working corridor leading to Kambin’s triangle, with the aim of simplifying access. Our previous study reported that TTP improved the efficiency of working cannula placement and shortened operative time during transarticular PE-LIF [11]. However, the available evidence on TTP remains largely limited to technical feasibility and short-term outcomes. Therefore, we retrospectively analyzed data from patients with single-level LDD who underwent TTP-assisted PE-LIF and had a minimum follow-up of 24 months to evaluate the midterm clinical, radiographic, and safety outcomes of the procedure.

## 2. Materials and Methods

### 2.1. Study Design

This retrospective cohort study included patients with single-level lumbar degenerative disease (LDD) who underwent transarticular trephonic plasty (TTP)-assisted percutaneous endoscopic lumbar interbody fusion (PE-LIF) at our institution between March 2020 and December 2023. All procedures were performed by one lead surgeon with first and second assistants. Before the study, the lead surgeon had performed approximately 54 PE-LIF procedures using conventional corridor creation. The TTP technique and channel version remained unchanged during the study. All patients underwent comprehensive preoperative imaging, including radiography, computed tomography (CT), and magnetic resonance imaging (MRI), and complete outcome records were available for the final analyzed cohort. The imaging equipment used included a New Oriental 1000FC digital radiography system (Beijing Wandong Medical Technology Co., Ltd., Beijing, China), a Brilliance iCT 256 CT scanner (Philips Medical Systems, Best, The Netherlands), and an Ingenia CX 3.0 T MRI scanner (Philips Medical Systems Nederland B.V., Best, The Netherlands). The data collection cutoff was 19 March 2026. The study was conducted in accordance with the Declaration of Helsinki and was approved by the Ethics Committee of Fuyang People’s Hospital, Fuyang, Anhui, China (approval No. [2024]201; 30 December 2024), covering retrospective use of clinical and imaging records. During follow-up, all patients provided written informed consent for the use of their de-identified clinical and imaging data for research purposes.

The present cohort is an expanded series with extended follow-up of 30 of the 39 TTP-treated patients in our previous publication [11], plus 100 additional patients. The previous study reported perioperative outcomes and visual analog scale (VAS) and Oswestry Disability Index (ODI) scores through 12 months. The present analysis adds longer follow-up, Japanese Orthopaedic Association (JOA) and 36-Item Short Form Health Survey (SF-36) scores, serial radiographic measurements, and final CT fusion assessment. Nine previously reported TTP-treated patients did not meet the present follow-up or data-completeness criteria.

### 2.2. Patient Selection

The inclusion criteria were as follows: (1) single-level LDD requiring surgical treatment with interbody fusion, including lumbar spinal stenosis, Meyerding grade I–II degenerative lumbar spondylolisthesis, or lumbar disc herniation with segmental instability; (2) concordance of the symptoms and clinical signs with the imaging findings at the index level, without substantial symptomatic relief after at least 3 months of standardized conservative treatment; (3) treatment with TTP-assisted PE-LIF; and (4) a follow-up duration of at least 24 months, with completion of the clinical and radiographic assessments at the prespecified time points. The exclusion criteria were as follows: (1) previous surgery at the index level; (2) disease involving two or more levels; (3) concomitant spinal infection, tumor, or marked structural spinal deformity; and (4) missing key clinical or imaging data.

Fusion was selected for symptomatic index-level stenosis with segmental instability, grade I–II degenerative spondylolisthesis, or disc herniation with instability. Standing flexion–extension lateral radiographs were used to measure dynamic sagittal translation and segmental angular change; instability was defined as translation of at least 3 mm or angular change of at least 10°. These diagnoses were pooled because they shared an indication for single-level decompression and instrumented interbody fusion. Baseline characteristics were also described by diagnosis. Exclusions were applied sequentially for clinical or surgical criteria, follow-up shorter than 24 months, and missing key outcome data; the three exclusion groups were mutually exclusive.

### 2.3. Surgical Technique

All procedures were performed under general anesthesia with the patient in the prone position. Neural decompression and discectomy were performed using a uniportal endoscopic technique with an iLESSYS Delta system (joimax GmbH, Karlsruhe, Germany). The pedicles at the index level were localized under C-arm fluoroscopy (Ziehm Vision RFD 3D; Ziehm Imaging GmbH, Nuremberg, Germany), and their surface projections were marked. An approximately 1.5 cm longitudinal incision was made lateral to each pedicle projection. After the fascia was incised, blunt finger dissection was performed through the intermuscular plane to the herringbone ridge. Four pedicle access needles and guidewires for cannulated pedicle screw placement were inserted under fluoroscopic guidance. The distal ends of the guidewires were secured away from the endoscopic operative field, and the incision over the caudal pedicle on the decompression side was then extended proximally from approximately 1.5 cm to a total length of approximately 2 cm.

Preoperative CT was used to plan the entry point and trajectory according to facet anatomy, bone quality, and available corridor width. Through the extended incision, a 3.0 mm Kirschner wire (K-wire) was anchored in the articular process at an abduction angle of approximately 15–20°. Anteroposterior fluoroscopy confirmed that the K-wire was positioned in the superolateral quadrant of the articular process, whereas lateral fluoroscopy confirmed that its trajectory was parallel to the target intervertebral space. Under lateral fluoroscopic guidance, the K-wire was advanced by tapping until it just penetrated the ventral cortex of the articular process. Sequential dilating guide rods and a soft-tissue dilation cannula were then advanced over the K-wire. Using the K-wire as the central axis, a hollow trephine with a 12 mm inner diameter, within a protective sleeve, was used to perform a one-step circumferential en bloc resection of the articular process, with the resection depth similarly controlled so that the trephine just penetrated the ventral cortex. The K-wire, guide rod, trephine, and resected bone block were subsequently removed as a single assembly, completing the TTP procedure. A guide rod and a beveled endoscopic working cannula were then inserted through the created osseous working corridor to reach Kambin’s triangle directly and expose the ligamentum flavum within the nerve root canal.

Advancement depth was controlled by repeated anteroposterior and lateral fluoroscopy, guide-rod markings, and tactile feedback at cortical breakthrough. No mechanical depth stop or intraoperative neuromonitoring was used. An eccentric guide permitted small adjustments for anchoring deviation. An off-target trajectory or unexpected advancement prompted cessation, repeat imaging, and repositioning; no planned TTP trajectory was abandoned in this cohort. After corridor creation, the exiting and traversing roots were identified and decompressed under direct endoscopic visualization.

Full-endoscopic decompression of the target region was performed according to the preoperative plan. In patients with central canal or contralateral lateral recess compression, bilateral decompression was performed through a unilateral approach using the over-the-top technique [12]. The nucleus pulposus and cartilaginous endplates were subsequently removed through Kambin’s triangle to prepare the interbody graft bed. Local autologous bone was packed into the intervertebral space through a bone graft funnel.

After decompression and disc-space preparation, sequential dilators restored disc height; the secondary dilator measured approximately 10 × 10 mm. A paired-blade cage-insertion channel had an approximately 10.5 mm inner dimension when closed. Temporary transverse expansion was controlled by the surgeon, usually within 3 mm and never exceeding 5 mm, and released after cage passage. Previously identified and decompressed neural structures were separated from the cage pathway by the medial blade; fluoroscopy confirmed bony position and insertion depth. A Fule Poster-series polyetheretherketone (PEEK) cage (FRP08; Beijing Fule Science & Technology Development Co., Ltd., Beijing, China; length 22–26 mm, width 10 mm, height 10–14 mm) was inserted with local autologous bone. Cage dimensions and lordotic angle were selected intraoperatively from the available options.

**Figure 1:**
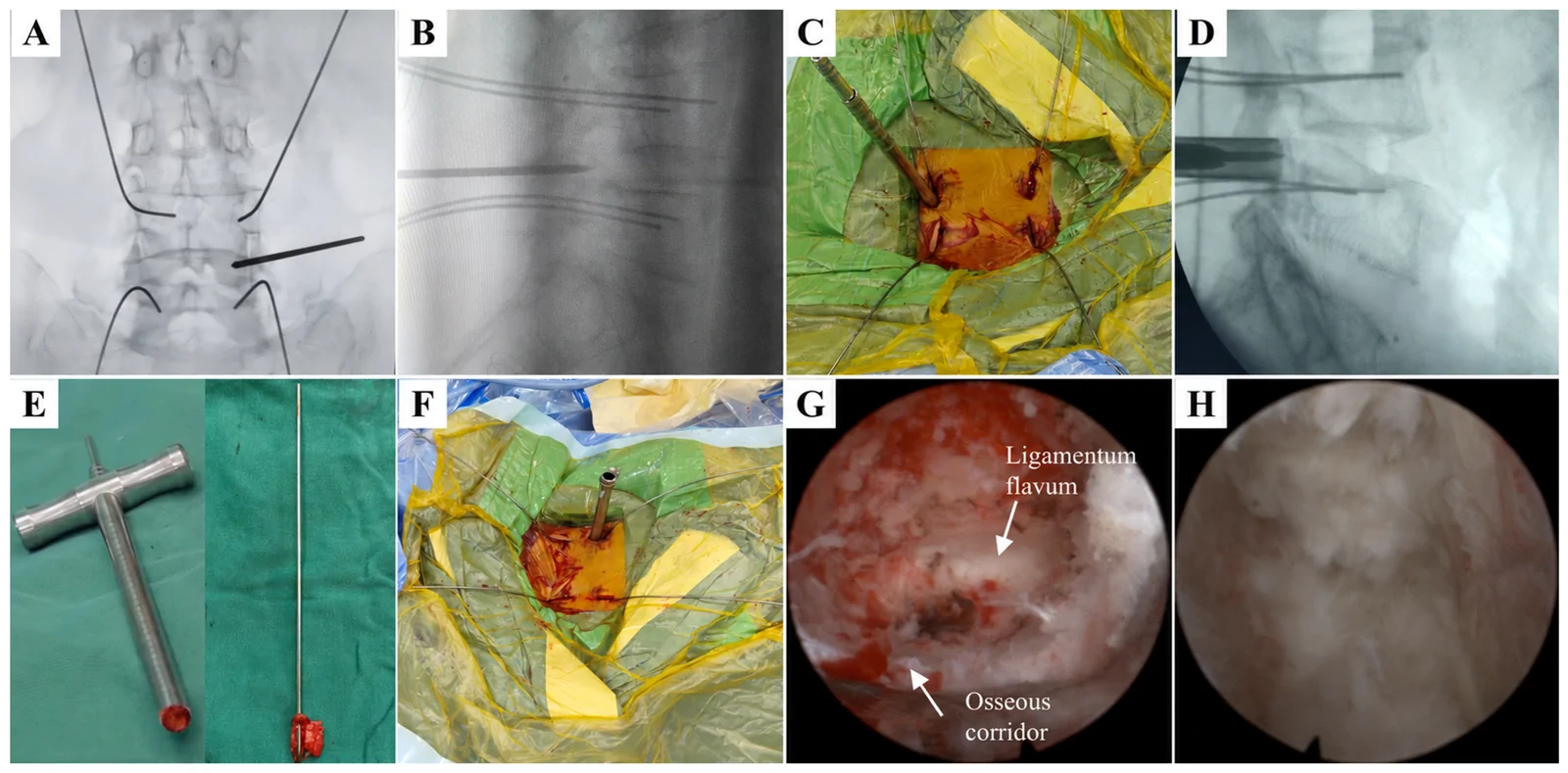
Key steps of transarticular trephonic plasty (TTP) during TTP-assisted percutaneous endoscopic lumbar interbody fusion (PE-LIF). The Kirschner wire (K-wire) was inserted through the superolateral quadrant of the articular process at an abduction angle of 15–20°, parallel to the target disc space, and advanced just beyond the ventral cortex of the articular process. Anteroposterior fluoroscopy (**A**) showing the K-wire tip at the lateral margin of the spinal canal. Lateral fluoroscopy (**B**) showing the K-wire parallel to the disc space with the tip just beyond the ventral cortex of the articular process. (**C**) Sequential soft-tissue dilation along the K-wire. (**D**) Coaxial advancement of a 12 mm trephine over the K-wire until ventral cortical breach. (**E**) En bloc retrieval of the K-wire, guide rod, trephine, and cylindrical bone block, completing creation of the osseous working corridor. (**F**) Direct insertion of the endoscopic working cannula through the osseous working corridor. (**G**) Initial endoscopic view showing the osseous corridor and ligamentum flavum within the nerve root canal. Arrows in panel G indicate the ligamentum flavum and osseous corridor. (**H**) Endoscopic view after intervertebral space preparation through Kambin’s triangle.

Repeat endoscopic inspection and fluoroscopic imaging confirmed adequate neural decompression and satisfactory cage position. Four percutaneous pedicle screws were subsequently placed over the guidewires, and the connecting rods were installed. The incisions were irrigated and closed in layers. The key operative steps of TTP-assisted PE-LIF are shown in Figure 1.

Postoperatively, vital signs and sensory and motor function of both lower extremities were closely monitored. Prophylactic antibiotics were routinely administered for 48 h, and anti-edema therapy, analgesia, and fluid replacement were provided as needed. Patients began ambulation on postoperative day 2 while wearing a soft lumbar support belt and were advised to avoid bending and strenuous weight-bearing activities for 3 months after surgery. All patients underwent regular outpatient follow-up.

### 2.4. Baseline and Perioperative Data Collection

The baseline characteristics recorded for all patients were age, sex, body mass index (BMI), symptom duration, primary diagnosis, surgical level, height, weight, smoking and alcohol history, diabetes, hypertension, bone health, American Society of Anesthesiologists (ASA) grade, and preoperative neurological findings. Perioperative variables included operative time, postoperative length of hospital stay, preoperative and postoperative day 1 creatine kinase (CK) levels, preoperative and postoperative day 1 hematocrit (HCT), and estimated total blood loss (eTBL).

Bone health was assessed by lumbar dual-energy X-ray absorptiometry using a Prodigy Primo scanner (GE Healthcare, Chicago, IL, USA). In postmenopausal women and men aged at least 50 years, normal bone density, low bone mass, and osteoporosis were defined by T scores of at least −1.0, between −2.5 and −1.0, and at most −2.5, respectively. In premenopausal women and younger men, Z scores above −2.0 denoted bone density within the expected range for age, and scores of at most −2.0 denoted density below that range; osteoporosis required a documented clinical diagnosis rather than the Z score alone.

Preoperative HCT and CK were obtained from routine preoperative blood tests, and postoperative samples were collected at approximately 07:00 on postoperative day 1. Fluids were given according to clinical needs during fasting, anesthesia, and postoperative care, without deliberate hemodilution. No patient received a perioperative transfusion or tranexamic acid. Individual fluid volumes were not used to correct the HCT-derived estimate.

The eTBL was calculated using the Nadler–Gross method. Predicted blood volume (PBV) was first calculated using the sex-specific Nadler equations [13]:

For men, PBV (L) = 0.3669 × [height (m)]^3^ + 0.03219 × weight (kg) + 0.6041.

For women, PBV (L) = 0.3561 × [height (m)]^3^ + 0.03308 × weight (kg) + 0.1833.

PBV was then converted from liters to milliliters (1 L = 1000 mL), and eTBL was estimated using the Gross equation [14]:

eTBL (mL) = PBV (mL) × (preoperative HCT − postoperative day 1 HCT) / mean HCT,

where mean HCT = (preoperative HCT + postoperative day 1 HCT) / 2.

The eTBL therefore represents an estimate based on the preoperative-to-day-1 HCT change, rather than directly measured intraoperative bleeding or cumulative blood loss over the entire postoperative course.

Complications occurring during the perioperative and follow-up periods were recorded. Reoperation was defined as any additional surgical procedure related to the index surgery and performed because of a complication or treatment failure. Adverse events were ascertained from inpatient records, outpatient assessments, imaging, and follow-up nurse contacts that included questions about care or operations elsewhere. Cage migration was defined as displacement of at least 3 mm from the immediate postoperative position, subsidence as at least 2 mm of endplate penetration, and implant loosening as a radiolucent line of at least 1 mm or a halo around a screw. Events were classified as symptomatic or radiographic without related symptoms. Radiographic fusion failure was reported separately from complications. Final clinical and fusion assessments included the treatment course after any revision procedure.

### 2.5. Clinical and Radiographic Outcomes

Clinical and radiographic evaluations were performed preoperatively, at an early postoperative assessment, at 6 and 12 months, and at final follow-up. The preoperative assessment was the most recent valid evaluation during the preoperative admission. Early VAS, JOA, and imaging assessments were obtained before discharge; ODI and SF-36 were assessed at approximately 1 month. The 6- and 12-month visits used predefined windows of ±4 weeks. Final follow-up was the last complete clinical and imaging assessment at least 24 months after surgery.

Clinical outcomes included separate back and leg pain VAS scores, the Chinese ODI, the Chinese JOA score, SF-36 Physical Component Summary (PCS) and Mental Component Summary (MCS) scores, and modified MacNab outcomes. VAS used a 10-cm horizontal line anchored by no pain and the worst imaginable pain. Patients marked the line, and distance from the zero anchor was measured in millimeters and converted to a 0–10 score with one decimal place. The 10 ODI items were scored from 0 to 5 and expressed as the total divided by 50 and multiplied by 100. JOA scores ranged from 0 to 29; final recovery rate was calculated separately for each patient as (final JOA − preoperative JOA)/(29 − preoperative JOA) × 100% [15,16]. MacNab outcomes were categorized as excellent, good, fair, or poor [17].

SF-36 used the Chinese version with the standard 4-week recall period. Its eight domains were recoded to 0–100 scales, and PCS and MCS were calculated using the version-specific summary scoring procedure consistently across visits. Questionnaires were collected by trained follow-up personnel; JOA and MacNab were assessed by spine surgeons other than the index operating surgeon. Assessments were recorded contemporaneously during routine follow-up and subsequently extracted for this study. Records with missing or non-applicable items were considered incomplete; no item-level imputation was performed.

Standing radiographs and MRI were available for all 130 included patients at all five imaging visits. CT was available in all patients preoperatively, before discharge, and at final follow-up, and selectively in 23 patients at 6 months and 32 at 12 months (Table S10). Examinations followed a standardized departmental follow-up protocol coordinated by a nurse and supported by research funding; they were not added for this retrospective analysis. CT assessed bone anatomy, implant position, and fusion. To limit radiation exposure, intermediate CT was reserved for persistent or worsening symptoms, suspected complications, abnormalities on other imaging, or a clinical need to assess fusion.

The radiographic parameters included lumbar lordosis (LL), defined as the Cobb angle between the tangents to the superior endplates of L1 and S1; segmental lordosis (SL), defined as the angle between the tangents to the superior endplate of the cranial vertebra and the inferior endplate of the caudal vertebra at the index level; disc angle (DA), defined as the angle between the tangents to the inferior endplate of the cranial vertebra and the superior endplate of the caudal vertebra at the index level; and disc height (DH), defined as the mean of the anterior, middle, and posterior disc heights at the index level. LL, SL, DA, and DH were measured on standing lateral lumbar radiographs. Foraminal height (FH) and foraminal area (FA) were measured on sagittal MR images at the index level on the side where the TTP corridor was created. FH was defined as the shortest distance between the inferior border of the pedicle of the cranial vertebra and the superior border of the pedicle of the caudal vertebra, and FA was traced within the superior and inferior pedicles, posterior vertebral/disc boundary anteriorly, and remaining articular-process boundary posteriorly. After transarticular resection, the anterior contour of the residual superior articular process defined the posterior boundary. Dural sac cross-sectional area (DCSA) was measured at the narrowest preoperative axial MRI section. Subsequent measurements used the corresponding anatomical level identified from adjacent endplates and pedicles, rather than selecting a new narrowest section at each visit.

At final follow-up, interbody fusion was evaluated on 1 mm lumbar CT images with sagittal, coronal, and axial reconstructions according to the Bridwell grading system. Grade I required continuous remodeled trabecular bridging; grade II required an intact graft without an interface radiolucent line but incomplete remodeling; grade III denoted an intact graft with an interface radiolucent line; and grade IV denoted nonunion with graft resorption or collapse. Grades I and II were defined as successful fusion, whereas grades III and IV were defined as fusion failure [18]. The two readers were independent of the operating team and blinded to clinical outcomes. All continuous radiographic parameters were independently measured by two spine surgeons using IntelliSpace PACS (version 4.4; Philips Medical Systems, Best, The Netherlands), and the mean of the two measurements was used for analysis. Bridwell grades were independently assigned by the two surgeons, and discrepant grades were adjudicated by a third senior spine surgeon.

Representative CT reconstructions used for fusion assessment are provided in Figure S2. Original paired measurements and pre-adjudication grades were retained for reliability analysis, linked by anonymous patient identifiers.

### 2.6. Statistical Analysis

Statistical analyses were performed in R (version 4.3.2; R Foundation for Statistical Computing, Vienna, Austria) using the nlme (version 3.1-163) and irr (version 0.84.1) packages. Continuous variables were summarized as means ± standard deviations (SDs), and categorical variables as counts and percentages. Follow-up duration was also summarized by the median, interquartile range (IQR), and range. Longitudinal analyses included the 130 patients with complete observations for all 13 outcomes at the five visits. ODI was selected as the main clinical outcome for this post hoc analysis; the remaining outcomes were exploratory.

A separate linear mixed-effects model with a patient-specific random intercept was fitted for each outcome. Visit was a categorical fixed effect with the preoperative assessment as the reference. Variation in final follow-up duration was accounted for by a covariate equal to actual final follow-up months minus 43.03 at the final visit and zero at other visits. This covariate estimated the association between outcome values and elapsed follow-up within the final category. Six- and 12-month observations were assigned to their predefined visit categories. Models were fitted by restricted maximum likelihood. Overall visit effects were assessed with marginal Wald *F* tests. Model-derived mean changes in the original units were reported with pointwise 95% confidence intervals (CIs); final-visit contrasts refer to 43.03 months.

Holm adjustment was applied separately to 13 overall visit tests, 52 baseline-versus-postoperative contrasts, and 39 adjacent postoperative contrasts. Adjacent-visit comparisons were exploratory. Model checks included residual Q–Q plots, residual distributions and variance patterns, and Shapiro–Wilk tests of normalized residuals. Sensitivity models allowed residual variance to differ by visit. Observed preoperative-to-final mean changes were also assessed using 10,000 patient-level bootstrap samples.

The included cohort was compared descriptively with the 45 patients excluded for missing key data using Welch tests, Fisher exact tests, and absolute standardized mean differences (SMDs), with unadjusted *p* values. Baseline characteristics were summarized by diagnosis; outcome changes by diagnosis were descriptive. Incomplete longitudinal records from excluded patients were not available for mixed-effects analysis, and no outcome imputation was performed.

Two-way absolute-agreement intraclass correlation coefficients (ICCs) with 95% CIs were calculated separately at each imaging visit for single readings, ICC(A, 1), and the mean of two readings, ICC(A, 2). Each analysis included 130 paired readings. Bland–Altman bias was the mean difference between the first and second observers, with limits of agreement equal to bias ± 1.96 SD of paired differences. Bridwell agreement before adjudication used linearly weighted Cohen kappa; unweighted kappa was a sensitivity measure. Kappa CIs used 10,000 patient-level bootstrap samples.

An exploratory responder analysis applied the thresholds described by Ostelo et al.: a decrease of at least 10 ODI points and at least 15 mm for each pain VAS, equivalent to 1.5 points on the recorded 0–10 scale [19]. Patients meeting the threshold, improving below it, remaining unchanged, or deteriorating were counted separately. Proportions, including fusion, MacNab, complication, and reoperation rates, were accompanied by exact Clopper–Pearson 95% CIs. Tests were two-sided, with adjusted *p* < 0.05 considered statistically significant for the longitudinal analyses.

## 3. Results

### 3.1. Baseline Characteristics and Perioperative Outcomes

A total of 254 patients underwent TTP-assisted PE-LIF during the study period. Four patients were first excluded for multilevel fusion (1) or previous index-level surgery (3), leaving 250. Of these, 75 had follow-up shorter than 24 months, leaving 175; a further 45 lacked key outcome data, and 130 were included (Figure S1). The final analysis included 130 patients: 48 men (36.9%) and 82 women (63.1%). The mean age was 66.77 ± 7.86 years, the mean body mass index was 24.65 ± 3.91 kg/m^2^, and the mean symptom duration was 36.25 ± 33.28 months. The primary diagnoses were lumbar spinal stenosis in 53 patients (40.8%), degenerative lumbar spondylolisthesis in 42 (32.3%), and lumbar disc herniation with segmental instability in 35 (26.9%). The surgical level was L3–4 in 8 patients (6.2%), L4–5 in 112 (86.2%), and L5–S1 in 10 (7.7%). All patients completed at least 24 months of follow-up, with a mean follow-up duration of 43.03 ± 9.03 months. Median follow-up was 42.5 months (IQR, 36.0–50.75; range, 29–60).

The primary reasons for follow-up shorter than 24 months were self-reported recovery and declining further follow-up (34), unsuccessful repeated contact (18), distance, work, or other access constraints (20), comorbidity or limited mobility (1), and death before the minimum follow-up interval (2). Each patient was assigned one primary reason. All 45 data-related exclusions involved missing imaging: 32 lacked imaging alone and 13 lacked both imaging and complete clinical assessments. Missingness affected 26 patients at 6 months, 29 at 12 months, and 36 at final follow-up, with overlap across visits and outcomes (Tables S8 and S9).

The included cohort and the 45 patients excluded for missing key data are compared in Table S1. Continuous-variable SMDs were at most 0.129. Baseline characteristics and descriptive outcome changes by diagnosis are shown in Table S2. Among the 42 patients with degenerative spondylolisthesis, 35 had grade I and 7 grade II. Preoperative motor weakness, sensory disturbance, abnormal reflexes, and cauda equina symptoms occurred in 33, 53, 37, and 1 patients, respectively; findings could overlap, and 84 patients (64.6%) had at least one of these findings. Decompression was unilateral in 92 patients, bilateral through a unilateral approach in 30, and through bilateral approaches in 8.

All 130 procedures were completed without conversion to open surgery. The mean operative time was 133.45 ± 15.63 min, and the mean postoperative length of hospital stay was 3.54 ± 0.67 days. The mean CK levels were 76.54 ± 15.91 IU/L preoperatively and 363.15 ± 28.31 IU/L on postoperative day 1, whereas the corresponding HCT values were 39.81 ± 3.48% and 36.78 ± 3.16%, respectively. The mean eTBL was 323.76 ± 66.24 mL. The baseline characteristics and perioperative outcomes are summarized in Table 1. The paired mean increase in CK was 286.61 ± 20.66 IU/L. No patient received a perioperative transfusion.

### 3.2. Clinical Outcomes

Holm-adjusted pairwise comparisons showed that the VAS scores for low back and leg pain and the ODI were significantly lower at every postoperative assessment than at baseline, whereas the JOA, PCS, and MCS scores were significantly higher (all adjusted *p* < 0.05). Pain scores decreased between the early postoperative assessment and 6 months. ODI decreased and JOA, PCS, and MCS increased through 12 months; PCS increased further from 12 months to final follow-up by 1.63 points (95% CI, 0.72–2.54; adjusted *p* = 0.010). Other final-versus-12-month clinical contrasts were not statistically significant.

**Figure 2:**
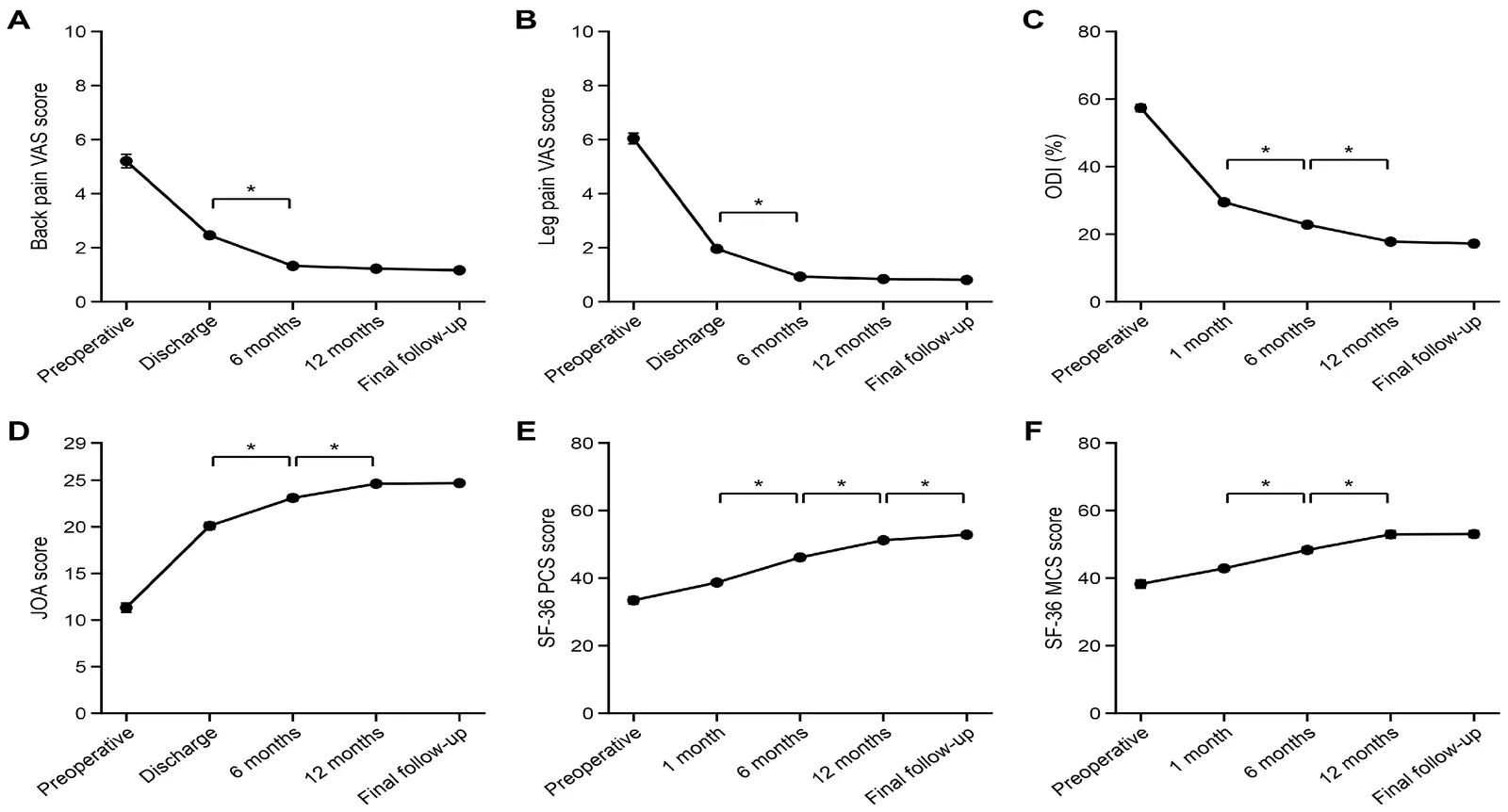
Longitudinal changes in clinical outcomes during follow-up. (**A**) Back pain VAS, (**B**) leg pain VAS, (**C**) ODI, (**D**) JOA, (**E**) SF-36 PCS, and (**F**) SF-36 MCS. Points show observed means and error bars show 95% CIs for 130 patients at each visit. Early VAS and JOA were measured before discharge; early ODI and SF-36 were measured at approximately 1 month. Brackets with asterisks indicate significant adjacent postoperative contrasts from linear mixed-effects models (Holm-adjusted *p* < 0.05 across 39 contrasts). All postoperative-versus-preoperative contrasts were significant after Holm adjustment across 52 comparisons. Final follow-up is a variable visit interval rather than a fixed postoperative month. Abbreviations: VAS = visual analog scale; ODI = Oswestry Disability Index; JOA = Japanese Orthopaedic Association score; SF-36 = 36-Item Short Form Health Survey; PCS = Physical Component Summary; MCS = Mental Component Summary; CI = confidence interval.

At the final follow-up, the mean VAS scores for low back and leg pain decreased from 5.20 ± 1.44 and 6.04 ± 1.13 preoperatively to 1.16 ± 0.39 and 0.81 ± 0.47, respectively. The mean ODI decreased from 57.35 ± 6.14 to 17.20 ± 2.80. The mean JOA score increased from 11.33 ± 2.85 to 24.69 ± 0.94, and the mean JOA recovery rate, calculated separately for each patient, was 75.36 ± 5.24%. The mean PCS and MCS scores increased from 33.43 ± 6.26 and 38.25 ± 6.87 to 52.83 ± 4.80 and 53.02 ± 5.59, respectively. At the final follow-up, modified MacNab outcomes were rated as excellent in 89 patients (68.5%), good in 30 (23.1%), fair in 7 (5.4%), and poor in 4 (3.1%). A total of 119 patients had excellent or good outcomes, yielding an excellent/good rate of 91.5% (95% CI, 85.4–95.7%). Longitudinal changes in the clinical outcomes are presented in Table 2 and Figure 2.

The estimated final-versus-preoperative ODI change was −40.15 points (95% CI, −41.02 to −39.29). Corresponding back and leg pain VAS changes were −4.04 (95% CI, −4.20 to −3.88) and −5.23 (95% CI, −5.36 to −5.10) points. All 130 patients met the ODI and leg pain improvement thresholds (100%; 95% CI, 97.2–100%); 127 met the back pain threshold (97.7%; 95% CI, 93.4–99.5%), and 3 improved below it. No patient had unchanged or worse final ODI or VAS scores.

Overall model tests are reported in Table S11, baseline and adjacent-visit contrasts in Tables S3 and S4, and diagnostic and sensitivity results in Table S6. ODI normalized residuals had a Shapiro–Wilk *W* of 0.997 (*p* = 0.193). Models allowing visit-specific residual variances and patient-level bootstrap estimates retained the direction of all preoperative-to-final mean changes.

### 3.3. Radiographic Outcomes

All radiographic measurements were higher at postoperative visits than at baseline (all Holm-adjusted *p* < 0.05). Between discharge and 6 months, LL, SL, DA, DH, FH, and FA decreased, whereas DCSA increased. SL decreased further from 6 to 12 months by 0.33° (95% CI, −0.53 to −0.14; adjusted *p* = 0.017). No final-versus-12-month radiographic contrast was statistically significant.

**Figure 3:**
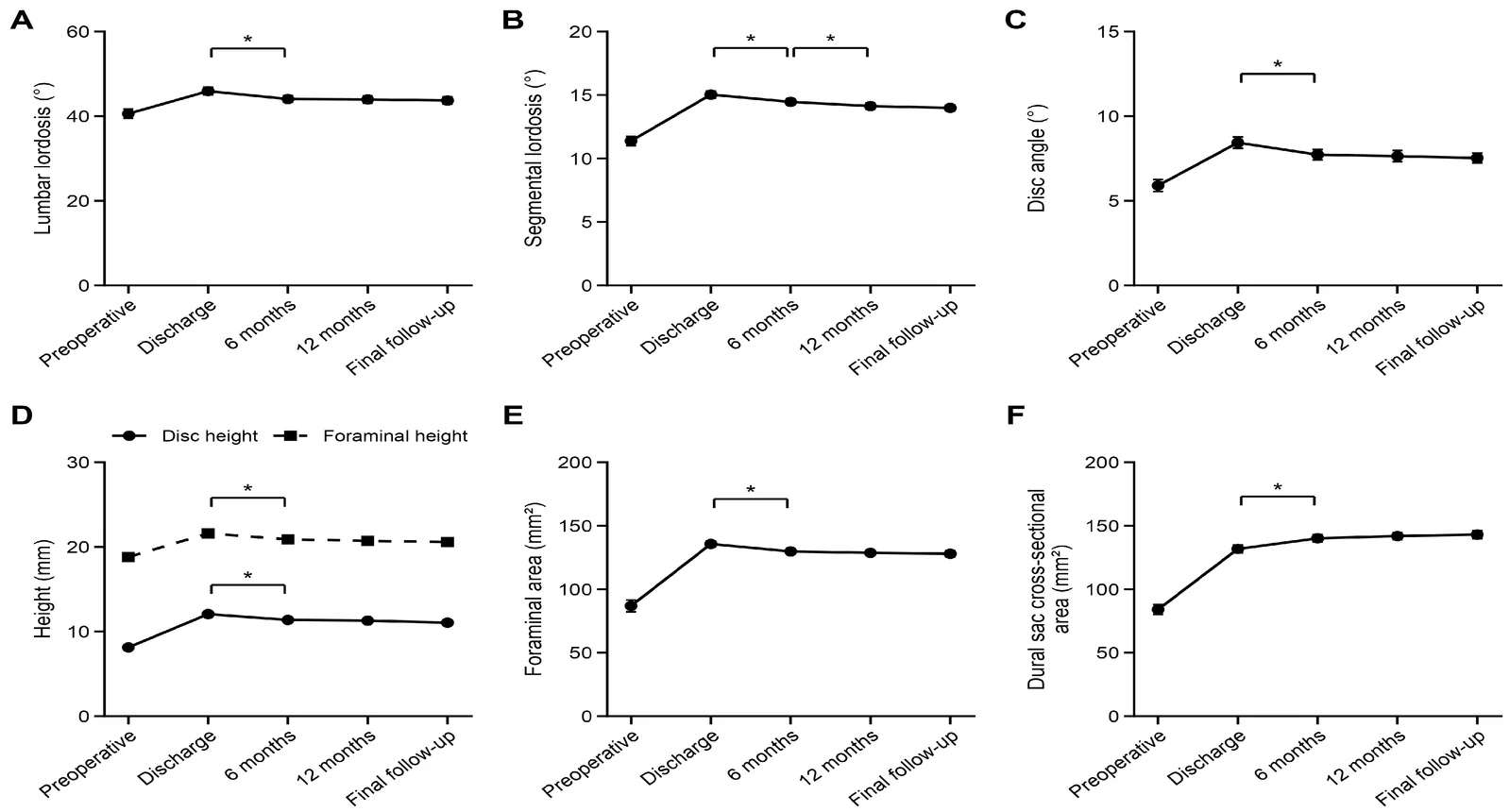
Longitudinal changes in radiographic parameters during follow-up. (**A**) Lumbar lordosis, (**B**) segmental lordosis, (**C**) disc angle, (**D**) disc height and foraminal height, (**E**) foraminal area, and (**F**) dural sac cross-sectional area. Points show observed means and error bars show 95% CIs for 130 patients at each visit. In panel (**D**), circles and solid lines indicate disc height; squares and dashed lines indicate foraminal height. Brackets with asterisks indicate significant adjacent postoperative contrasts from linear mixed-effects models (Holm-adjusted *p* < 0.05 across 39 contrasts). All postoperative-versus-preoperative contrasts were significant after Holm adjustment across 52 comparisons. Early imaging was obtained before discharge. Final follow-up is a variable visit interval.

At the final follow-up, the mean LL, SL, and DA increased from preoperative values of 40.57 ± 6.37°, 11.38 ± 2.09°, and 5.90 ± 2.04° to 43.70 ± 4.79°, 13.98 ± 1.31°, and 7.53 ± 1.70°, respectively. The mean DH, FH, FA, and DCSA increased from 8.12 ± 1.63 mm, 18.80 ± 2.42 mm, 86.85 ± 26.73 mm^2^, and 84.00 ± 21.94 mm^2^ preoperatively to 11.05 ± 1.39 mm, 20.57 ± 2.07 mm, 127.93 ± 13.58 mm^2^, and 143.02 ± 18.14 mm^2^, respectively. Longitudinal changes in the radiographic parameters are presented in Table 3 and Figure 3.

**Figure 4:**
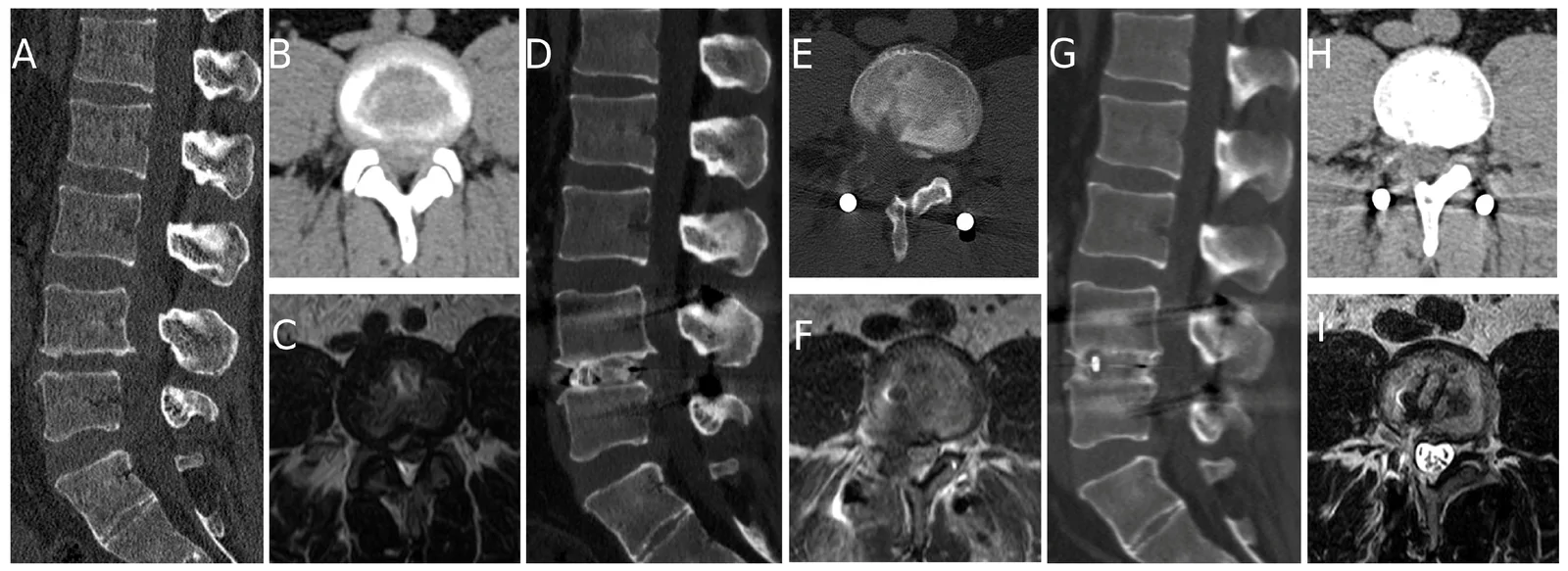
Representative perioperative radiographic images after TTP-assisted PE-LIF. Images are shown for a 46-year-old male patient with chronic L4–5 central lumbar disc herniation, severe dural sac compression, and recent progressive cauda equina symptoms. He had received at least 3 months of conservative treatment. Flexion–extension radiographs showed 3.5 mm dynamic L4–5 translation and an 8° angular change; fusion was selected for index-level instability accompanying the compressive disc herniation. The patient underwent TTP-assisted PE-LIF. (**A**–**C**) Preoperative CT sagittal, CT axial, and MRI axial images; (**D**–**F**) CT sagittal, CT axial, and MRI axial images obtained at discharge; (**G**–**I**) final follow-up CT sagittal, CT axial, and MRI axial images. CT = computed tomography; MRI = magnetic resonance imaging; PE-LIF = percutaneous endoscopic lumbar interbody fusion; TTP = transarticular trephonic plasty.

Final follow-up CT showed Bridwell grade I fusion in 75 patients (57.7%), grade II in 43 (33.1%), grade III in 9 (6.9%), and grade IV in 3 (2.3%). Bridwell grade I or II fusion was observed in 118 patients, yielding a fusion rate of 90.8% (95% CI, 84.4–95.1%). The final clinical and radiographic endpoints are summarized in Table 4, and representative preoperative and final follow-up images are shown in Figure 4.

All 11 fair or poor MacNab outcomes occurred in patients with grade III or IV fusion findings; one additional grade III patient had an excellent MacNab outcome. The 12 grade III/IV patients were managed with observation and conservative care because there was no progressive neurological or radicular deterioration, hardware failure, cage migration, or clinically important subsidence requiring revision. Final clinical and fusion outcomes included assessments after revision surgery where applicable.

Visit-specific single-reading ICCs ranged from 0.751 to 0.810, and ICCs for the mean of two readings from 0.858 to 0.895. Bridwell agreement before adjudication was 94.6% (123/130), with weighted kappa of 0.923 (95% CI, 0.856–0.973) and unweighted kappa of 0.902 (95% CI, 0.823–0.966). Full ICCs and Bland–Altman limits are reported in Table S5.

### 3.4. Safety Outcomes

Complications occurred in 10 patients, yielding an overall patient-level complication rate of 7.7% (95% CI, 3.8–13.7%). Two patients underwent reoperation, corresponding to a reoperation rate of 1.5% (95% CI, 0.2–5.4%). No spinal epidural hematoma or permanent neurological injury occurred.

**Figure 5:**
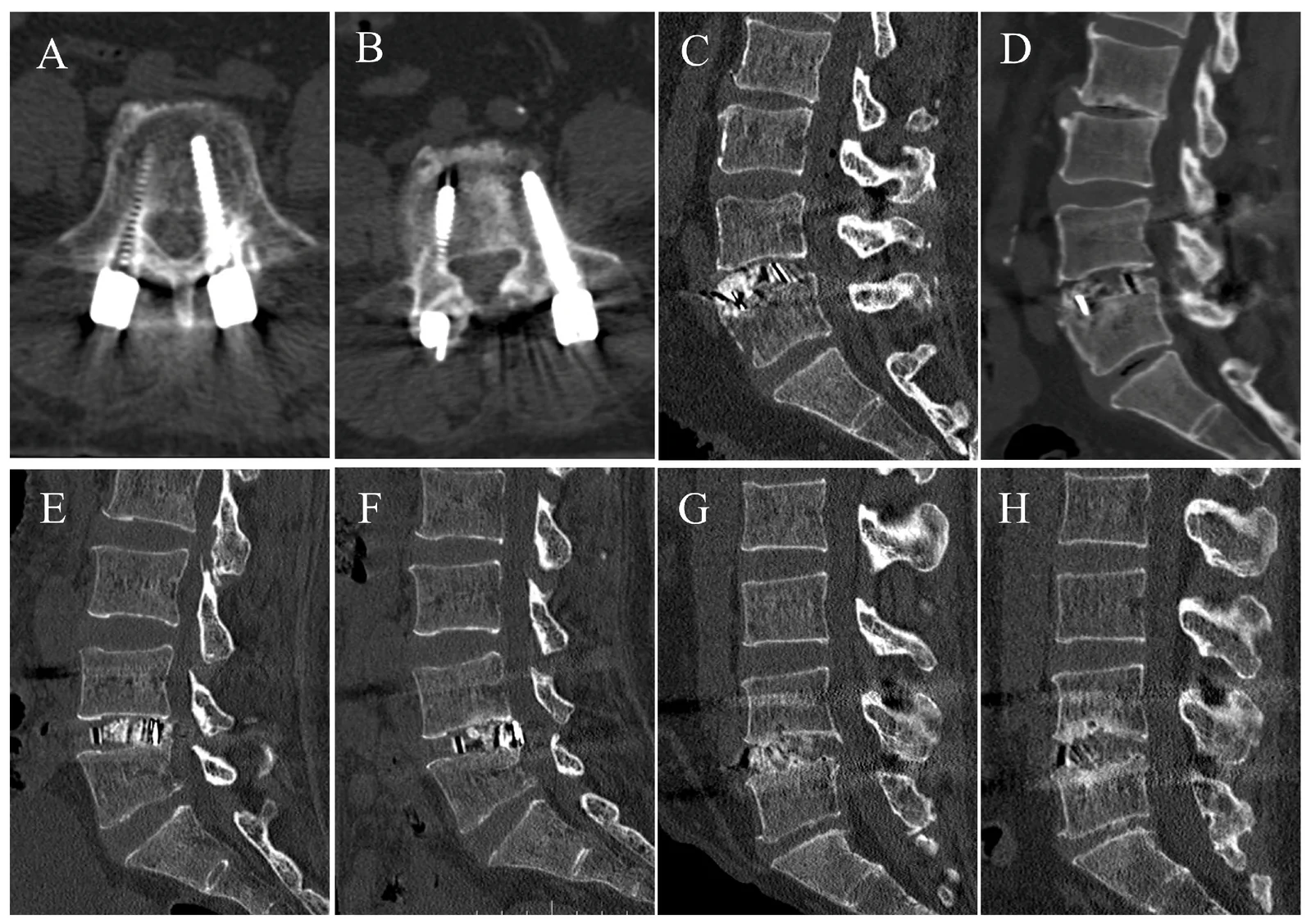
Representative imaging of complications and unfavorable radiographic outcomes after TTP-assisted PE-LIF. (**A**,**B**) Axial CT images of a 61-year-old male patient with L4–5 lumbar spinal stenosis showing medial breach of the left L5 pedicle screw into the spinal canal; the screw position was corrected after revision of the spinal fixation construct, with complete resolution of radicular pain. (**C**,**D**) Sagittal CT images of a 73-year-old male patient with Meyerding grade I L4 spondylolisthesis showing anterior cortical and superior endplate fracture of the L5 vertebral body; final follow-up CT showed satisfactory healing of the anterior cortical fracture and interbody bone graft. (**E**,**F**) Sagittal CT images of a 66-year-old female patient with L4–5 lumbar disc herniation showing satisfactory initial fixation and cage position, followed by posterior cage migration during follow-up without apparent related clinical symptoms. (**G**,**H**) Sagittal CT images of a 47-year-old male patient with L4–5 lumbar spinal stenosis showing adequate immediate postoperative interbody grafting and subsequent graft resorption with absence of solid interbody fusion at final follow-up. CT = computed tomography; PE-LIF = percutaneous endoscopic lumbar interbody fusion; TTP = transarticular trephonic plasty.

The complications included two dural tears; following meticulous fascial closure, neither patient developed wound leakage or secondary pseudomeningocele. One patient developed ipsilateral motor weakness (grade 4) and one developed ipsilateral sensory loss after discectomy; strength recovered to grade 5 and sensation normalized at 2 months after neurotrophic therapy and observation. Two superficial incisional infections resolved with dressing changes and antibiotic therapy. Symptomatic pedicle screw malposition was identified in two patients; both developed radicular pain on postoperative day 1, underwent CT confirmation and screw revision that day, with complete resolution of radicular pain. One patient sustained a fracture involving the anterior vertebral cortex and superior endplate during cage insertion; the fracture subsequently healed with conservative treatment. Another patient developed radiographic cage migration but remained asymptomatic throughout follow-up. Patient-level event timing, manifestations, management, revision status, and outcome are provided in Table S7. The 10 complication patients did not overlap with the 12 patients with grade III/IV fusion findings. No additional operations or adverse events treated elsewhere were recorded. The safety outcomes are also summarized in Table 4, and representative images of the complications and adverse radiographic findings are shown in Figure 5.

## 4. Discussion

Percutaneous endoscopic lumbar interbody fusion (PE-LIF), performed using a large-channel spinal endoscopic platform, enables neural decompression, endplate preparation, and interbody fusion under direct endoscopic visualization while limiting soft-tissue injury [7,20]. The transarticular approach provides a shorter access route to Kambin’s triangle and facilitates broader contralateral decompression and harvesting of local autologous bone [21]. However, when establishing working access, conventional uniportal PE-LIF often requires repeated soft-tissue exposure, hemostasis, localization, and incremental bone resection. Transarticular trephonic plasty (TTP) was designed to simplify this critical step. The present study evaluated the midterm outcomes of TTP-assisted PE-LIF for LDD. Over a mean follow-up of 43.03 months, the cohort showed sustained clinical improvement and maintained radiographic correction, with complications in 7.7% and reoperation in 1.5% of analyzed patients.

A previous study reported shorter working-cannula placement and total operative times with TTP than with conventional access [11]. Thirty patients overlap with the present cohort, so the current series extends and expands those observations. The previous study also reported more fluoroscopic exposures during cannula placement with TTP (10.5 ± 4.4 versus 1.3 ± 1.5); these were exposure counts for corridor creation, not total-procedure radiation dose. Fluoroscopic exposure was not recorded in the present cohort. Two pedicle-edge fractures during TTP in the previous study were excluded here because follow-up was shorter than 24 months. They were distinct from the current fracture during cage insertion and remain relevant to procedural risk. Operative times vary across published endoscopic fusion studies [22,23]; comparisons with the present mean of 133.45 ± 15.63 min provide context rather than evidence of comparative efficiency. En bloc bone removal may simplify corridor creation, although its independent effect on total operative burden was not measured in this series.

During endoscopic surgery, continuous irrigation makes visible intraoperative blood loss difficult to quantify [24], and visible bleeding does not include all hidden loss [25]. The Nadler–Gross calculation used here yielded an estimated 323.76 ± 66.24 mL from the preoperative-to-day-1 HCT change [13,14]. This estimate is sensitive to dilution and fluid redistribution and does not describe the entire postoperative blood-loss course. Blood sampling and tranexamic acid protocols also affect comparisons between studies [25,26]. CK increased from 76.54 ± 15.91 to 363.15 ± 28.31 IU/L on day 1, with a paired mean increase of 286.61 ± 20.66 IU/L. Published endoscopic fusion studies assess postoperative CK at different intervals [27,28]. Our single postoperative measurement describes an early biochemical response, so neither CK nor HCT-derived eTBL can be taken as evidence of reduced muscle injury or bleeding from TTP.

Clinical improvements were maintained through midterm follow-up. At the group level, back and leg pain scores decreased between the early assessment and 6 months, whereas ODI, JOA, and SF-36 scores changed through 12 months, with a further PCS increase at final follow-up. This pattern is limited by the assessment intervals and different early questionnaire timing; nonsignificant later contrasts do not establish a recovery plateau or equivalence. The final mean JOA recovery rate was 75.36 ± 5.24%, and 119 patients had excellent or good MacNab outcomes. Earlier PE-LIF studies provide context for these improvements [29,30]. The high ODI and VAS responder proportions describe the selected cohort with complete follow-up. Meeting an improvement threshold does not exclude a complication, persistent limitation, or radiographic nonunion, as illustrated by the 11 fair or poor MacNab outcomes among patients with grade III/IV fusion findings.

Direct decompression removes compressive bone, disc, and ligamentous tissue. Disc-height restoration can also change the dimensions of the canal and neural foramen [31,32,33,34]. In this cohort, average DH, FH, and FA remained above baseline at final follow-up despite reductions from their early postoperative values. Foraminal measurements were made on the TTP side, and direct removal of the posterior boundary contributes to the measured area change; this cannot be assigned solely to disc-space restoration. DCSA increased after discharge. Resolution of early postoperative edema or hematoma and subsequent remodeling are possible explanations [35,36,37]; we could not separate their individual contributions. A maintained average disc height does not, by itself, exclude subsidence in an individual patient.

Lumbar lordosis, segmental lordosis, and disc angle were higher at final follow-up than preoperatively. No patient-specific alignment target was evaluated, so these findings describe the direction of change rather than achievement of an optimal sagittal correction. Final CT showed grade I fusion in 75 patients (57.7%) and grade II in 43 (33.1%); the composite grade I/II rate was 90.8%. The distinction matters because grade II represents an intact graft without interface lucency but incomplete remodeling. Published fusion rates after other procedures are descriptive context [38,39,40], given differences in patients, imaging, follow-up, and fusion criteria. Endplate preparation and local autologous bone were components of the procedure [20,21]. Cage dimensions and material may affect interface loading [41,42,43,44]; such effects were not tested in this cohort.

Complications occurred in 10 patients (7.7%; 95% CI, 3.8–13.7%), and 2 required reoperation (1.5%; 95% CI, 0.2–5.4%). The event types have been reported after lumbar interbody fusion [45,46], but retrospective ascertainment and selection of patients with complete follow-up constrain comparisons. Operative records located dural and neurological events during discectomy, screw malposition during fixation, and an anterior cortical/superior endplate fracture during cage insertion. No lower risk during corridor creation can be inferred from this distribution. The previous pedicle-edge fractures demonstrate that TTP itself can cause osseous injury [11]. Accurate CT planning, fluoroscopic depth control, and direct visualization of neural structures after corridor creation remain essential. Ligamentum flavum may provide an anatomical buffer [10,11], but does not assure protection. The cage-insertion fracture also supports careful control of expansion, cage size, and depth; its precise mechanism was not established.

This study has several limitations. Because the design was single-center, retrospective, and single-arm, the effects of TTP cannot be separated from decompression, grafting, cage insertion, and fixation. Only 130 of 254 operated patients entered the complete-data cohort. Comparison with 45 patients excluded for missing data cannot establish the representativeness of the 75 patients with insufficient follow-up, and imaging-driven exclusions may select patients with different outcomes. Diagnostic pooling reflects common fusion indications but does not establish equivalent outcomes across diagnoses. ODI was selected as the main outcome post hoc, and the numerous other outcomes and adjacent comparisons remain exploratory despite multiplicity adjustment. The model accounts for variation within the final follow-up category but does not estimate a continuous postoperative trajectory; incompletely observed excluded patients could not be included. All procedures were performed by one experienced lead surgeon; reproducibility and the learning curve remain unevaluated. Perioperative blood loss and muscle injury are only approximated by HCT-derived eTBL and a single day-1 CK measurement. Some retrospective imaging bias remains despite blinded independent reading and formal reliability estimates. Prospective multicenter studies with concurrent comparison groups are needed to assess comparative outcomes and risks.

## 5. Conclusions

TTP-assisted PE-LIF was associated with sustained clinical improvement in the analyzed single-level degenerative lumbar disease cohort. Final grade I/II fusion, complication, and reoperation rates were 90.8%, 7.7%, and 1.5%, respectively. These findings describe the complete surgical treatment and follow-up course; controlled studies are needed to determine the independent contribution of TTP.

## Figures and Tables

**Table 1:** Baseline characteristics and perioperative outcomes.

Characteristic	Value
No. of patients	130
**Demographic characteristics**	
Age, years	66.77 ± 7.86
Sex	
Male	48 (36.9)
Female	82 (63.1)
Height, m	1.64 ± 0.08
Weight, kg	66.37 ± 10.69
BMI, kg/m^2^	24.65 ± 3.91
Symptom duration, months	36.25 ± 33.28
Follow-up duration, months	43.03 ± 9.03
Follow-up median (IQR), months	42.5 (36.0–50.75)
Follow-up range, months	29–60
Smoking history	51 (39.2)
Alcohol history	20 (15.4)
Diabetes	22 (16.9)
Hypertension	25 (19.2)
**Bone health**	
Bone density within normal/expected range	39 (30.0)
Low bone mass/below expected range	50 (38.5)
Osteoporosis	41 (31.5)
**ASA classification**	
ASA grade I	9 (6.9)
ASA grade II	120 (92.3)
ASA grade III	1 (0.8)
**Primary diagnosis**	
Lumbar spinal stenosis	53 (40.8)
Degenerative lumbar spondylolisthesis	42 (32.3)
Meyerding grade I (spondylolisthesis)	35 (83.3)
Meyerding grade II (spondylolisthesis)	7 (16.7)
Lumbar disc herniation with segmental instability	35 (26.9)
**Preoperative neurological findings**	
Motor weakness	33 (25.4)
Sensory disturbance	53 (40.8)
Abnormal reflexes	37 (28.5)
Cauda equina symptoms	1 (0.8)
At least one of these neurological findings	84 (64.6)
**Surgical level**	
L3–4	8 (6.2)
L4–5	112 (86.2)
L5–S1	10 (7.7)
**Decompression approach**	
Unilateral decompression	92 (70.8)
Bilateral decompression via unilateral approach	30 (23.1)
Bilateral decompression via bilateral approaches	8 (6.2)
**Perioperative outcomes**	
Operative time, min	133.45 ± 15.63
Postoperative hospital stay, days	3.54 ± 0.67
Preoperative CK, IU/L	76.54 ± 15.91
Postoperative day 1 CK, IU/L	363.15 ± 28.31
CK change (day 1—preoperative), IU/L	286.61 ± 20.66
Preoperative HCT, %	39.81 ± 3.48
Postoperative day 1 HCT, %	36.78 ± 3.16
Estimated total blood loss (eTBL), mL	323.76 ± 66.24

Legend: Values are means ± SD or *n* (%), with median (IQR) and range also provided for follow-up duration. Meyerding percentages use the 42 spondylolisthesis patients; other percentages use 130. Neurological findings may overlap. Estimated blood loss uses the preoperative-to-day-1 HCT change. Bone-health categories follow the DXA criteria in Section 2.4. Bold indicates category headings. Abbreviations: SD = standard deviation; BMI = body mass index; CK = creatine kinase; eTBL = estimated total blood loss; HCT = hematocrit. ASA = American Society of Anesthesiologists; DXA = dual-energy X-ray absorptiometry; IQR = interquartile range.

**Table 2:** Longitudinal changes in clinical outcomes during follow-up.

Variable	Preoperative	Early Postoperative	6 Months	12 Months	Final Follow-Up	Adjusted *p*
VAS back pain	5.20 ± 1.44	2.46 ± 0.48	1.32 ± 0.38 *	1.22 ± 0.38	1.16 ± 0.39	<0.001
VAS leg pain	6.04 ± 1.13	1.95 ± 0.54	0.93 ± 0.40 *	0.84 ± 0.48	0.81 ± 0.47	<0.001
ODI, %	57.35 ± 6.14	29.45 ± 4.67	22.80 ± 4.16 *	17.77 ± 3.89 *	17.20 ± 2.80	<0.001
JOA score	11.33 ± 2.85	20.10 ± 2.13	23.10 ± 1.80 *	24.62 ± 1.64 *	24.69 ± 0.94	<0.001
SF-36 PCS	33.43 ± 6.26	38.69 ± 4.06	46.12 ± 4.30 *	51.20 ± 4.56 *	52.83 ± 4.80 *	<0.001
SF-36 MCS	38.25 ± 6.87	42.88 ± 4.61	48.33 ± 5.54 *	52.93 ± 6.17 *	53.02 ± 5.59	<0.001

Legend: Values are means ± SD for 130 patients at each visit. *p* values refer to overall categorical visit effects in linear mixed-effects models, with Holm adjustment across 13 outcomes. All postoperative-versus-preoperative contrasts were significant after Holm adjustment across 52 comparisons. * *p* < 0.05 versus the preceding postoperative visit after Holm adjustment across 39 exploratory comparisons. Models included a patient-specific random intercept and adjustment for final follow-up duration. Early VAS and JOA were assessed before discharge; early ODI and SF-36 were assessed at approximately 1 month. Mean changes and 95% CIs are reported in Tables S3 and S4. Abbreviations: SD = standard deviation; VAS = visual analog scale; ODI = Oswestry Disability Index; JOA = Japanese Orthopaedic Association score; SF-36 = 36-Item Short Form Health Survey; PCS = Physical Component Summary; MCS = Mental Component Summary. CI = confidence interval.

**Table 3:** Longitudinal changes in radiographic parameters during follow-up.

Variable	Preoperative	Discharge	6 Months	12 Months	Final Follow-Up	Adjusted *p*
LL, °	40.57 ± 6.37	45.91 ± 5.17	44.05 ± 4.51 *	43.94 ± 4.84	43.70 ± 4.79	<0.001
SL, °	11.38 ± 2.09	15.03 ± 1.67	14.46 ± 1.34 *	14.12 ± 1.45 *	13.98 ± 1.31	<0.001
DA, °	5.90 ± 2.04	8.43 ± 1.92	7.72 ± 1.75 *	7.64 ± 1.90	7.53 ± 1.70	<0.001
DH, mm	8.12 ± 1.63	12.06 ± 1.39	11.37 ± 1.44 *	11.28 ± 1.33	11.05 ± 1.39	<0.001
FH, mm	18.80 ± 2.42	21.59 ± 2.35	20.89 ± 2.32 *	20.71 ± 2.30	20.57 ± 2.07	<0.001
FA, mm^2^	86.85 ± 26.73	135.64 ± 12.50	129.75 ± 13.06 *	128.65 ± 12.47	127.93 ± 13.58	<0.001
DCSA, mm^2^	84.00 ± 21.94	131.73 ± 18.41	140.09 ± 16.83 *	141.87 ± 16.35	143.02 ± 18.14	<0.001

Legend: Values are means ± SD for 130 patients at each visit. *p* values refer to overall categorical visit effects in linear mixed-effects models, with Holm adjustment across 13 outcomes. All postoperative-versus-preoperative contrasts were significant after Holm adjustment across 52 comparisons. * *p* < 0.05 versus the preceding postoperative visit after Holm adjustment across 39 exploratory comparisons. Models included a patient-specific random intercept and adjustment for final follow-up duration. Mean changes and 95% CIs are reported in Tables S3 and S4. Abbreviations: SD = standard deviation; LL = lumbar lordosis; SL = segmental lordosis; DA = disc angle; DH = disc height; FH = foraminal height; FA = foraminal area; DCSA = dural sac cross-sectional area.

**Table 4:** Final clinical and radiographic outcomes and safety summary.

Variable	Value	95% CI, %
No. of patients	130	
**Interbody fusion status (CT-based Bridwell grade)**		
Grade I	75 (57.7)	
Grade II	43 (33.1)	
Grade III	9 (6.9)	
Grade IV	3 (2.3)	
Fusion rate (Grades I and II)	118 (90.8)	84.4–95.1
**Modified MacNab outcome at final follow-up**		
Excellent	89 (68.5)	
Good	30 (23.1)	
Fair	7 (5.4)	
Poor	4 (3.1)	
Excellent/good rate	119 (91.5)	85.4–95.7
**Safety outcomes**		
Overall complication rate	10 (7.7)	3.8–13.7
Reoperation rate	2 (1.5)	0.2–5.4
Clinically important improvement at final follow-up		
ODI decrease ≥ 10 points	130 (100.0)	97.2–100
Back pain VAS decrease ≥ 1.5 points	127 (97.7)	93.4–99.5
Leg pain VAS decrease ≥ 1.5 points	130 (100.0)	97.2–100
Back pain improvement below threshold	3 (2.3)	0.5–6.6
Unchanged ODI or VAS at final follow-up	0 (0.0)	
Worse ODI or VAS at final follow-up	0 (0.0)	

Legend: Values are presented as number (%). Successful fusion was defined as Bridwell grades I and II. Fusion status and modified MacNab outcomes were assessed at the final follow-up. Safety outcomes include perioperative and follow-up adverse events. Confidence intervals for proportions are exact 95% CIs. Radiographic grade III/IV fusion failure is separate from the complication count. Final clinical and fusion outcomes include the complete treatment course after revision. Bold indicates category headings. Abbreviation: CT = computed tomography.

## Data Availability

The de-identified participant-level clinical and imaging data supporting the findings of this study are available from the corresponding author, Wei Jiao (0558jw@163.com), upon reasonable request, subject to approval by the Ethics Committee of Fuyang People’s Hospital and compliance with applicable institutional requirements and patient-privacy regulations. To protect participant privacy, these data are not deposited in a publicly accessible repository.

## Supplementary Materials

The following supporting information can be downloaded at: https://www.mdpi.com/article/10.3390/jcm15207980/s1, Figure S1: Sequential patient selection. Exclusions were mutually exclusive; Figure S2: Representative postoperative CT reconstructions used for fusion assessment; Table S1: Included patients and patients excluded for missing key data; Table S2: Characteristics and descriptive outcomes by diagnosis; Table S3: Model-derived changes from baseline; Table S4: Exploratory adjacent postoperative contrasts; Table S5: Visit-specific observer agreement; Table S6: Residual checks and sensitivity estimates; Table S7: Patient-level adverse events; Table S8: Missing assessments in the 45 excluded patients; Table S9: Outcome-specific missingness among excluded patients; Table S10: Imaging examinations available in the analyzed cohort; Table S11: Overall visit effects in mixed-effects models.

## Abbreviations

The following abbreviations are used in this manuscript:

ASA	American Society of Anesthesiologists
BMI	body mass index
CI	confidence interval
CK	creatine kinase
CT	computed tomography
DA	disc angle
DCSA	dural sac cross-sectional area
DH	disc height
DXA	dual-energy X-ray absorptiometry
eTBL	estimated total blood loss
FA	foraminal area
FH	foraminal height
HCT	hematocrit
ICC	intraclass correlation coefficient
IQR	interquartile range
JOA	Japanese Orthopaedic Association
K-wire	Kirschner wire
LDD	lumbar degenerative disease
LL	lumbar lordosis
LMM	linear mixed-effects model
MCID	minimal clinically important difference
MCS	Mental Component Summary
MIS-TLIF	minimally invasive transforaminal lumbar interbody fusion
MRI	magnetic resonance imaging
ODI	Oswestry Disability Index
PBV	predicted blood volume
PCS	Physical Component Summary
PE-LIF	percutaneous endoscopic lumbar interbody fusion
PEEK	polyetheretherketone
SD	standard deviation
SF-36	36-Item Short Form Health Survey
SL	segmental lordosis
SMD	standardized mean difference
TLIF	transforaminal lumbar interbody fusion
TTP	transarticular trephonic plasty
UELIF	uniportal endoscopic lumbar interbody fusion
VAS	visual analog scale
