# Supplementary material for: Midterm Clinical and Radiographic Outcomes of Transarticular Trephonic Plasty-Assisted Percutaneous Endoscopic Lumbar Interbody Fusion for Single-Level Lumbar Degenerative Disease: A Retrospective Cohort Study
Source: J Clin Med. 2026 Oct 15;15(20):7980. doi: 10.3390/jcm15207980 (PMC13705862; doi:10.3390/jcm15207980)
Supplement: Supplementary file 1 [file jcm-15-07980-s001.zip › jcm-4587379-supplementary.pdf]

Supplementary Materials

Midterm Clinical and Radiographic Outcomes of Transarticular Trephonic Plasty-Assisted Percutaneous Endoscopic Lumbar Interbody Fusion for Single-Level Lumbar Degenerative Disease: A Retrospective Cohort Study

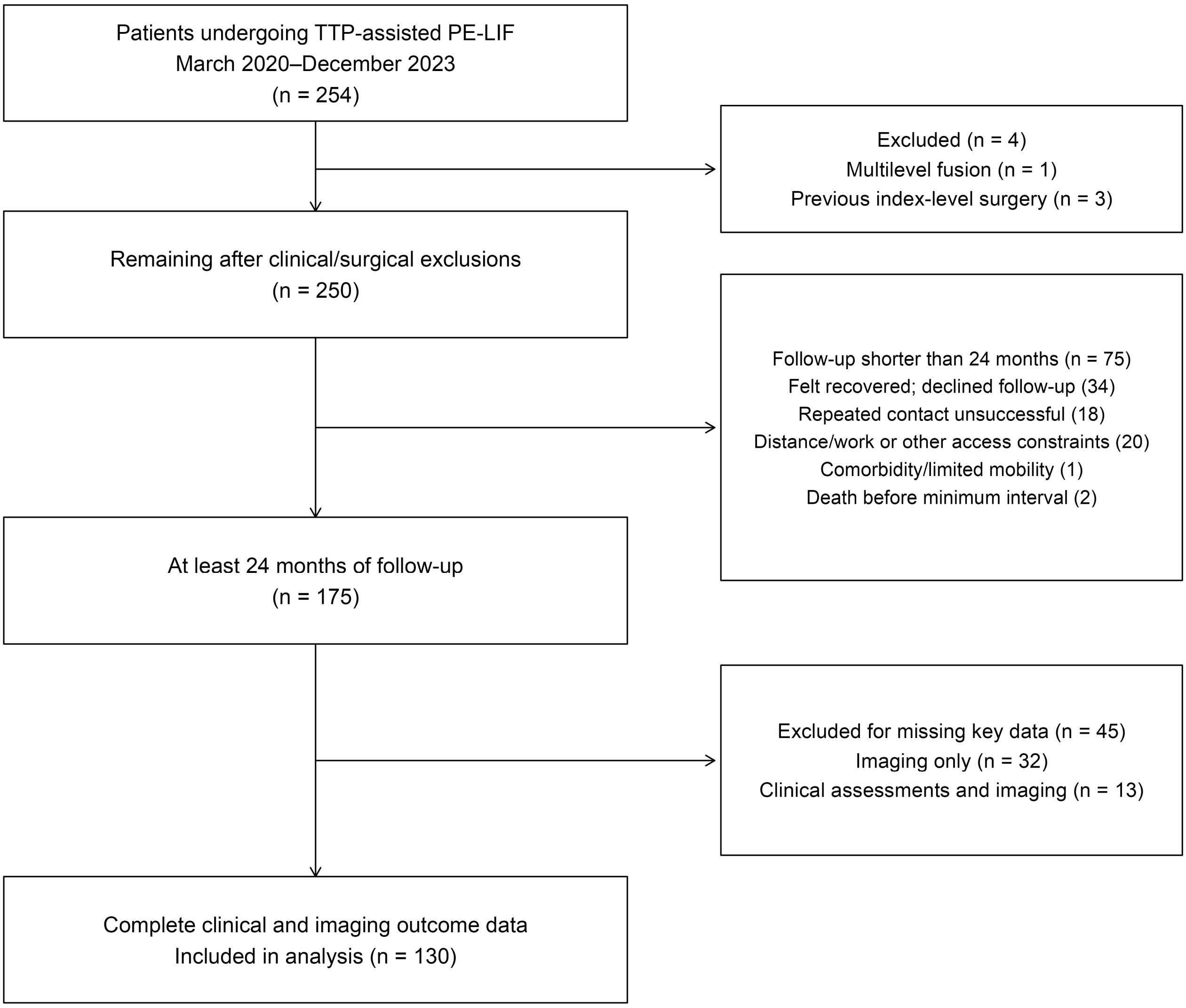

Figure S1. Sequential patient selection. Exclusions were mutually exclusive. Reasons for follow-up shorter than 24 months were assigned as one primary reason per patient. The data collection cutoff was 19 March 2026.

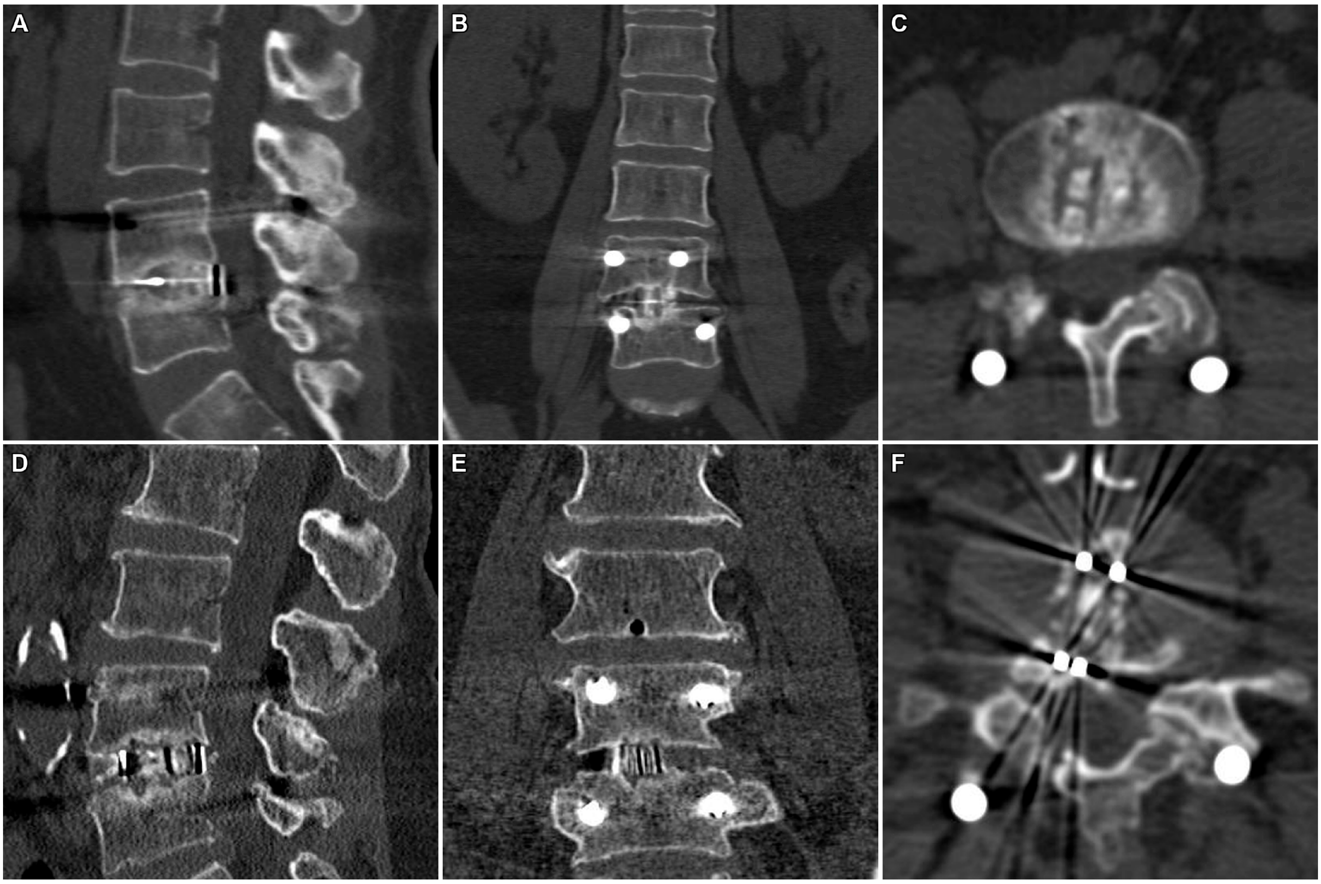

Figure S2. Representative postoperative CT reconstructions used for fusion assessment. Panels A–C show a patient with Bridwell grade I fusion, and panels D–F show a patient with Bridwell grade II fusion. Columns show sagittal, coronal, and axial reconstructions, respectively. Fusion grading was based on the full CT examination using the criteria in Section 2.5.

Table S1. Included patients and patients excluded for missing key data

Characteristic	Included (*n* = 130)	Excluded (*n* = 45)	Absolute SMD	*P*
Age, years	66.77 ± 7.86	67.09 ± 7.42	0.042	0.807
Symptom duration, months	36.25 ± 33.28	35.51 ± 33.41	0.022	0.898
BMI, kg/m²	24.65 ± 3.91	24.70 ± 3.94	0.012	0.945
Preoperative back pain VAS	5.20 ± 1.44	5.07 ± 1.48	0.089	0.612
Preoperative leg pain VAS	6.04 ± 1.13	6.12 ± 1.18	0.073	0.677
Preoperative ODI, %	57.35 ± 6.14	57.38 ± 6.39	0.004	0.983
Preoperative JOA	11.33 ± 2.85	11.27 ± 3.07	0.022	0.902
Preoperative SF-36 PCS	33.43 ± 6.26	32.74 ± 6.54	0.107	0.541
Preoperative SF-36 MCS	38.25 ± 6.87	38.52 ± 7.16	0.038	0.827
Preoperative LL, °	40.57 ± 6.37	40.99 ± 6.58	0.065	0.712
Preoperative SL, °	11.38 ± 2.09	11.13 ± 2.22	0.115	0.513
Preoperative DA, °	5.90 ± 2.04	6.04 ± 2.16	0.066	0.708
Preoperative DH, mm	8.12 ± 1.63	7.91 ± 1.71	0.127	0.470
Preoperative FH, mm	18.80 ± 2.42	18.61 ± 2.51	0.077	0.660
Preoperative FA, mm²	86.85 ± 26.73	84.19 ± 27.40	0.098	0.573
Preoperative DCSA, mm²	84.00 ± 21.94	81.14 ± 22.58	0.128	0.463

Characteristic	Included (*n* = 130)	Excluded (*n* = 45)	Absolute SMD	*P*
Operative time, min	133.45 ± 15.63	133.29 ± 15.39	0.011	0.951
Postoperative stay, days	3.54 ± 0.67	3.56 ± 0.69	0.023	0.896
Estimated blood loss, mL	323.76 ± 66.24	322.87 ± 67.87	0.013	0.940
Sex: Female	82 (63.1)	29 (64.4)	0.028	1.000
Sex: Male	48 (36.9)	16 (35.6)		
Diagnosis: Stenosis with instability	53 (40.8)	19 (42.2)	0.031	1.000
Diagnosis: Degenerative spondylolisthesis	42 (32.3)	14 (31.1)		
Diagnosis: Disc herniation with instability	35 (26.9)	12 (26.7)		
Level: L3-4	8 (6.2)	3 (6.7)	0.044	1.000
Level: L4-5	112 (86.2)	39 (86.7)		
Level: L5-S1	10 (7.7)	3 (6.7)		
Smoking history: No	79 (60.8)	27 (60.0)	0.016	1.000
Smoking history: Yes	51 (39.2)	18 (40.0)		
Alcohol history: No	110 (84.6)	38 (84.4)	0.005	1.000
Alcohol history: Yes	20 (15.4)	7 (15.6)		
Diabetes: No	108 (83.1)	37 (82.2)	0.023	1.000
Diabetes: Yes	22 (16.9)	8 (17.8)		
Hypertension: No	105 (80.8)	36 (80.0)	0.019	1.000
Hypertension: Yes	25 (19.2)	9 (20.0)		
Bone health: Normal/within expected range	39 (30.0)	13 (28.9)	0.033	1.000
Bone health: Low/below expected range	50 (38.5)	18 (40.0)		
Bone health: Osteoporosis	41 (31.5)	14 (31.1)		
ASA grade: I	9 (6.9)	3 (6.7)	0.120	0.616
ASA grade: II	120 (92.3)	41 (91.1)		
ASA grade: III	1 (0.8)	1 (2.2)		

Continuous variables: mean ± SD and Welch tests. Categorical variables: *n* (%) and Fisher exact tests. SMDs are absolute and comparisons descriptive, with unadjusted *P* values.

Table S2. Characteristics and descriptive outcomes by diagnosis

Characteristic	Stenosis (*n* = 53)	Spondylolisthesis (*n* = 42)	Disc herniation (*n* = 35)	*P*
Age, years	67.02 ± 7.35	67.02 ± 7.92	66.09 ± 8.70	0.855
BMI, kg/m²	24.98 ± 4.11	24.07 ± 3.10	24.85 ± 4.46	0.426
Symptom duration, months	42.43 ± 37.10	33.48 ± 28.92	30.23 ± 31.32	0.227
Follow-up duration, months	40.40 ± 9.76	44.76 ± 8.79	44.94 ± 7.23	0.027
Operative time, min	129.57 ± 13.61	136.33 ± 17.05	135.89 ± 15.92	0.054
Female	32 (60.4)	30 (71.4)	20 (57.1)	0.361
Male	21 (39.6)	12 (28.6)	15 (42.9)	
L3-4	4 (7.5)	2 (4.8)	2 (5.7)	0.650

Characteristic	Stenosis (*n* = 53)	Spondylolisthesis (*n* = 42)	Disc herniation (*n* = 35)	*P*
L4-5	47 (88.7)	36 (85.7)	29 (82.9)	
L5-S1	2 (3.8)	4 (9.5)	4 (11.4)	
Smoking history	20 (37.7)	20 (47.6)	11 (31.4)	0.340
Alcohol history	6 (11.3)	6 (14.3)	8 (22.9)	0.341
Diabetes	12 (22.6)	6 (14.3)	4 (11.4)	0.385
Hypertension	10 (18.9)	8 (19.0)	7 (20.0)	1.000
Normal/within expected range	12 (22.6)	15 (35.7)	12 (34.3)	0.554
Low/below expected range	24 (45.3)	15 (35.7)	11 (31.4)	
Osteoporosis	17 (32.1)	12 (28.6)	12 (34.3)	
ASA grade 1	4 (7.5)	5 (11.9)	0 (0.0)	0.088
ASA grade 2	49 (92.5)	36 (85.7)	35 (100.0)	
ASA grade 3	0 (0.0)	1 (2.4)	0 (0.0)	
Preoperative-to-final ODI change	−40.15 ± 6.01	−39.90 ± 5.53	−40.46 ± 5.72	
Bridwell grade I/II fusion	46 (86.8)	38 (90.5)	34 (97.1)	

All stenosis/disc-herniation patients had index-level instability. Values are mean ± SD or *n* (%). Baseline comparisons use Welch ANOVA or Fisher exact tests; *P* values are unadjusted. ODI changes and fusion proportions are descriptive, with no subgroup outcome hypothesis test.

Table S3. Model-derived changes from baseline

Outcome	Contrast	Change (95% CI)	Adjusted *P*
Back pain VAS	Early postoperative − Preoperative	−2.75 (−2.90 to −2.59)	< 0.001
Back pain VAS	6 months − Preoperative	−3.88 (−4.04 to −3.72)	< 0.001
Back pain VAS	12 months − Preoperative	−3.98 (−4.14 to −3.82)	< 0.001
Back pain VAS	Final follow-up − Preoperative	−4.04 (−4.20 to −3.88)	< 0.001
Leg pain VAS	Early postoperative − Preoperative	−4.08 (−4.21 to −3.95)	< 0.001
Leg pain VAS	6 months − Preoperative	−5.11 (−5.24 to −4.98)	< 0.001
Leg pain VAS	12 months − Preoperative	−5.20 (−5.33 to −5.07)	< 0.001
Leg pain VAS	Final follow-up − Preoperative	−5.23 (−5.36 to −5.10)	< 0.001
ODI	Early postoperative − Preoperative	−27.91 (−28.77 to −27.04)	< 0.001
ODI	6 months − Preoperative	−34.55 (−35.42 to −33.69)	< 0.001
ODI	12 months − Preoperative	−39.58 (−40.45 to −38.72)	< 0.001
ODI	Final follow-up − Preoperative	−40.15 (−41.02 to −39.29)	< 0.001
JOA	Early postoperative − Preoperative	8.77 (8.38 to 9.16)	< 0.001
JOA	6 months − Preoperative	11.77 (11.38 to 12.16)	< 0.001
JOA	12 months − Preoperative	13.29 (12.90 to 13.68)	< 0.001
JOA	Final follow-up − Preoperative	13.36 (12.97 to 13.75)	< 0.001
SF-36 PCS	Early postoperative − Preoperative	5.26 (4.36 to 6.17)	< 0.001
SF-36 PCS	6 months − Preoperative	12.70 (11.79 to 13.60)	< 0.001
SF-36 PCS	12 months − Preoperative	17.78 (16.87 to 18.68)	< 0.001
SF-36 PCS	Final follow-up − Preoperative	19.41 (18.50 to 20.31)	< 0.001
SF-36 MCS	Early postoperative − Preoperative	4.62 (3.60 to 5.65)	< 0.001

Outcome	Contrast	Change (95% CI)	Adjusted *P*
SF-36 MCS	6 months − Preoperative	10.07 (9.05 to 11.09)	< 0.001
SF-36 MCS	12 months − Preoperative	14.68 (13.66 to 15.70)	< 0.001
SF-36 MCS	Final follow-up − Preoperative	14.77 (13.75 to 15.79)	< 0.001
LL	Early postoperative − Preoperative	5.34 (4.64 to 6.03)	< 0.001
LL	6 months − Preoperative	3.48 (2.78 to 4.17)	< 0.001
LL	12 months − Preoperative	3.37 (2.67 to 4.06)	< 0.001
LL	Final follow-up − Preoperative	3.13 (2.43 to 3.82)	< 0.001
SL	Early postoperative − Preoperative	3.65 (3.46 to 3.85)	< 0.001
SL	6 months − Preoperative	3.08 (2.88 to 3.27)	< 0.001
SL	12 months − Preoperative	2.75 (2.55 to 2.94)	< 0.001
SL	Final follow-up − Preoperative	2.60 (2.41 to 2.80)	< 0.001
DA	Early postoperative − Preoperative	2.53 (2.31 to 2.76)	< 0.001
DA	6 months − Preoperative	1.82 (1.60 to 2.05)	< 0.001
DA	12 months − Preoperative	1.74 (1.51 to 1.96)	< 0.001
DA	Final follow-up − Preoperative	1.63 (1.40 to 1.85)	< 0.001
DH	Early postoperative − Preoperative	3.94 (3.78 to 4.09)	< 0.001
DH	6 months − Preoperative	3.25 (3.09 to 3.40)	< 0.001
DH	12 months − Preoperative	3.16 (3.01 to 3.32)	< 0.001
DH	Final follow-up − Preoperative	2.93 (2.77 to 3.08)	< 0.001
FH	Early postoperative − Preoperative	2.80 (2.51 to 3.08)	< 0.001
FH	6 months − Preoperative	2.10 (1.81 to 2.38)	< 0.001
FH	12 months − Preoperative	1.92 (1.63 to 2.20)	< 0.001
FH	Final follow-up − Preoperative	1.78 (1.49 to 2.06)	< 0.001
FA	Early postoperative − Preoperative	48.79 (46.36 to 51.23)	< 0.001
FA	6 months − Preoperative	42.90 (40.46 to 45.34)	< 0.001
FA	12 months − Preoperative	41.80 (39.36 to 44.24)	< 0.001
FA	Final follow-up − Preoperative	41.08 (38.64 to 43.52)	< 0.001
DCSA	Early postoperative − Preoperative	47.73 (45.54 to 49.93)	< 0.001
DCSA	6 months − Preoperative	56.08 (53.89 to 58.28)	< 0.001
DCSA	12 months − Preoperative	57.87 (55.67 to 60.06)	< 0.001
DCSA	Final follow-up − Preoperative	59.02 (56.82 to 61.21)	< 0.001

Random-intercept mixed-effects models with categorical visits and adjustment for final follow-up duration. Final contrasts refer to 43.03 months. CIs are pointwise 95% CIs; Holm adjustment covers 52 baseline contrasts. Changes use the original scale: degrees for angles, mm for heights, mm² for areas, and points for clinical scores. Each model uses 130 patients.

Table S4. Exploratory adjacent postoperative contrasts

Outcome	Contrast	Change (95% CI)	Adjusted *P*
Back pain VAS	6 months − Early postoperative	−1.13 (−1.29 to −0.97)	< 0.001
Back pain VAS	12 months − 6 months	−0.10 (−0.26 to 0.06)	1.000
Back pain VAS	Final follow-up − 12 months	−0.06 (−0.22 to 0.10)	1.000
Leg pain VAS	6 months − Early postoperative	−1.03 (−1.16 to −0.90)	< 0.001

Outcome	Contrast	Change (95% CI)	Adjusted *P*
Leg pain VAS	12 months − 6 months	−0.09 (−0.22 to 0.04)	1.000
Leg pain VAS	Final follow-up − 12 months	−0.03 (−0.16 to 0.10)	1.000
ODI	6 months − Early postoperative	−6.65 (−7.51 to −5.78)	< 0.001
ODI	12 months − 6 months	−5.03 (−5.90 to −4.17)	< 0.001
ODI	Final follow-up − 12 months	−0.57 (−1.43 to 0.30)	1.000
JOA	6 months − Early postoperative	3.00 (2.61 to 3.39)	< 0.001
JOA	12 months − 6 months	1.52 (1.13 to 1.91)	< 0.001
JOA	Final follow-up − 12 months	0.07 (−0.32 to 0.46)	1.000
SF-36 PCS	6 months − Early postoperative	7.43 (6.53 to 8.34)	< 0.001
SF-36 PCS	12 months − 6 months	5.08 (4.17 to 5.99)	< 0.001
SF-36 PCS	Final follow-up − 12 months	1.63 (0.72 to 2.54)	0.010
SF-36 MCS	6 months − Early postoperative	5.45 (4.43 to 6.47)	< 0.001
SF-36 MCS	12 months − 6 months	4.61 (3.58 to 5.63)	< 0.001
SF-36 MCS	Final follow-up − 12 months	0.09 (−0.93 to 1.11)	1.000
LL	6 months − Early postoperative	−1.86 (−2.56 to −1.16)	< 0.001
LL	12 months − 6 months	−0.11 (−0.80 to 0.59)	1.000
LL	Final follow-up − 12 months	−0.24 (−0.94 to 0.46)	1.000
SL	6 months − Early postoperative	−0.57 (−0.77 to −0.38)	< 0.001
SL	12 months − 6 months	−0.33 (−0.53 to −0.14)	0.017
SL	Final follow-up − 12 months	−0.14 (−0.34 to 0.05)	1.000
DA	6 months − Early postoperative	−0.71 (−0.94 to −0.49)	< 0.001
DA	12 months − 6 months	−0.09 (−0.31 to 0.14)	1.000
DA	Final follow-up − 12 months	−0.11 (−0.33 to 0.11)	1.000
DH	6 months − Early postoperative	−0.69 (−0.85 to −0.53)	< 0.001
DH	12 months − 6 months	−0.08 (−0.24 to 0.07)	1.000
DH	Final follow-up − 12 months	−0.24 (−0.39 to −0.08)	0.063
FH	6 months − Early postoperative	−0.70 (−0.98 to −0.42)	< 0.001
FH	12 months − 6 months	−0.18 (−0.46 to 0.10)	1.000
FH	Final follow-up − 12 months	−0.14 (−0.42 to 0.14)	1.000
FA	6 months − Early postoperative	−5.89 (−8.33 to −3.45)	< 0.001
FA	12 months − 6 months	−1.10 (−3.54 to 1.34)	1.000
FA	Final follow-up − 12 months	−0.72 (−3.16 to 1.71)	1.000
DCSA	6 months − Early postoperative	8.35 (6.16 to 10.55)	< 0.001
DCSA	12 months − 6 months	1.78 (−0.41 to 3.98)	1.000
DCSA	Final follow-up − 12 months	1.15 (−1.05 to 3.34)	1.000

Random-intercept mixed-effects models with categorical visits and adjustment for final follow-up duration. Final contrasts refer to 43.03 months. CIs are pointwise 95% CIs; Holm adjustment covers 39 exploratory adjacent contrasts. Changes use the original scale: degrees for angles, mm for heights, mm^2 for areas, and points for clinical scores. Each model uses 130 patients.

Table S5. Visit-specific observer agreement

Outcome	Visit	ICC(A, 1) (95% CI)	ICC(A, 2) (95% CI)	Limits of agreement
LL	Preoperative	0.800 (0.728 to 0.854)	0.889 (0.842 to 0.921)	−8.362 to 8.362

Outcome	Visit	ICC(A, 1) (95% CI)	ICC(A, 2) (95% CI)	Limits of agreement
LL	Early postoperative	0.798 (0.726 to 0.853)	0.888 (0.841 to 0.921)	−6.808 to 6.808
LL	6 months	0.789 (0.714 to 0.846)	0.882 (0.833 to 0.917)	−6.099 to 6.099
LL	12 months	0.799 (0.727 to 0.854)	0.888 (0.842 to 0.921)	−6.361 to 6.362
LL	Final follow-up	0.803 (0.732 to 0.856)	0.891 (0.845 to 0.923)	−6.228 to 6.228
SL	Preoperative	0.801 (0.729 to 0.855)	0.889 (0.844 to 0.922)	−2.735 to 2.735
SL	Early postoperative	0.775 (0.696 to 0.836)	0.873 (0.821 to 0.911)	−2.340 to 2.340
SL	6 months	0.790 (0.715 to 0.847)	0.882 (0.834 to 0.917)	−1.804 to 1.803
SL	12 months	0.756 (0.671 to 0.821)	0.861 (0.803 to 0.902)	−2.124 to 2.124
SL	Final follow-up	0.759 (0.676 to 0.824)	0.863 (0.806 to 0.903)	−1.900 to 1.900
DA	Preoperative	0.779 (0.701 to 0.839)	0.876 (0.824 to 0.912)	−2.832 to 2.832
DA	Early postoperative	0.775 (0.696 to 0.836)	0.873 (0.821 to 0.911)	−2.687 to 2.687
DA	6 months	0.783 (0.706 to 0.842)	0.878 (0.828 to 0.914)	−2.404 to 2.404
DA	12 months	0.810 (0.742 to 0.862)	0.895 (0.852 to 0.926)	−2.413 to 2.413
DA	Final follow-up	0.759 (0.675 to 0.823)	0.863 (0.806 to 0.903)	−2.477 to 2.476
DH	Preoperative	0.797 (0.724 to 0.852)	0.887 (0.840 to 0.920)	−2.154 to 2.153
DH	Early postoperative	0.774 (0.695 to 0.835)	0.873 (0.820 to 0.910)	−1.952 to 1.952
DH	6 months	0.765 (0.683 to 0.828)	0.867 (0.811 to 0.906)	−2.069 to 2.069
DH	12 months	0.760 (0.676 to 0.824)	0.863 (0.807 to 0.903)	−1.936 to 1.936
DH	Final follow-up	0.760 (0.676 to 0.824)	0.863 (0.807 to 0.903)	−2.019 to 2.019
FH	Preoperative	0.777 (0.699 to 0.837)	0.875 (0.823 to 0.911)	−3.374 to 3.374
FH	Early postoperative	0.790 (0.715 to 0.847)	0.883 (0.834 to 0.917)	−3.166 to 3.166
FH	6 months	0.807 (0.738 to 0.860)	0.893 (0.849 to 0.925)	−2.977 to 2.977
FH	12 months	0.754 (0.669 to 0.820)	0.860 (0.802 to 0.901)	−3.394 to 3.394
FH	Final follow-up	0.751 (0.665 to 0.817)	0.858 (0.799 to 0.900)	−3.068 to 3.068
FA	Preoperative	0.779 (0.701 to 0.839)	0.876 (0.824 to 0.912)	−37.043 to 37.043
FA	Early postoperative	0.777 (0.699 to 0.837)	0.875 (0.823 to 0.911)	−17.413 to 17.413
FA	6 months	0.771 (0.691 to 0.833)	0.871 (0.817 to 0.909)	−18.463 to 18.463
FA	12 months	0.754 (0.668 to 0.819)	0.860 (0.801 to 0.901)	−18.380 to 18.380
FA	Final follow-up	0.799 (0.727 to 0.854)	0.888 (0.842 to 0.921)	−17.847 to 17.847
DCSA	Preoperative	0.756 (0.671 to 0.821)	0.861 (0.803 to 0.902)	−32.207 to 32.207
DCSA	Early postoperative	0.764 (0.682 to 0.827)	0.866 (0.811 to 0.906)	−26.457 to 26.457
DCSA	6 months	0.802 (0.731 to 0.856)	0.890 (0.844 to 0.922)	−21.952 to 21.952
DCSA	12 months	0.771 (0.690 to 0.832)	0.871 (0.817 to 0.909)	−23.134 to 23.134
DCSA	Final follow-up	0.765 (0.684 to 0.828)	0.867 (0.812 to 0.906)	−26.001 to 26.001

Two-way absolute-agreement ICCs use 130 patient pairs per row. ICC(A, 1) is a single reading; ICC(A, 2) is the mean of two readings. Bias is first minus second observer; limits are bias ± 1.96 SD. Units match the corresponding imaging outcome. For Bridwell fusion grades at final follow-up, the two observers agreed in 123 of 130 patients (94.6%). Weighted kappa was 0.923 (95% CI, 0.856–0.973), and unweighted kappa was 0.902 (95% CI, 0.823–0.966).

Table S6. Residual checks and sensitivity estimates

Outcome	Shapiro–Wilk W	P	Visit-specific variance: final change (95% CI)	Observed paired bootstrap change (95% CI)
Back pain VAS	0.915	< 0.001	−4.04 (−4.27 to −3.80)	−4.04 (−4.27 to −3.81)
Leg pain VAS	0.986	< 0.001	−5.23 (−5.41 to −5.05)	−5.23 (−5.41 to −5.05)
ODI	0.997	0.193	−40.15 (−41.13 to −39.18)	−40.15 (−41.12 to −39.17)
JOA	0.974	< 0.001	13.36 (12.91 to 13.81)	13.36 (12.92 to 13.80)
SF-36 PCS	0.997	0.305	19.41 (18.34 to 20.47)	19.41 (18.34 to 20.48)
SF-36 MCS	0.996	0.077	14.77 (13.60 to 15.94)	14.77 (13.59 to 15.95)
LL	0.986	< 0.001	3.13 (2.25 to 4.01)	3.13 (2.16 to 4.10)
SL	0.994	0.008	2.60 (2.36 to 2.85)	2.60 (2.33 to 2.87)
DA	0.985	< 0.001	1.63 (1.38 to 1.88)	1.63 (1.35 to 1.91)
DH	0.986	< 0.001	2.93 (2.74 to 3.11)	2.93 (2.72 to 3.13)
FH	0.981	< 0.001	1.78 (1.46 to 2.09)	1.78 (1.42 to 2.13)
FA	0.939	< 0.001	41.08 (37.57 to 44.59)	41.08 (37.33 to 44.72)
DCSA	0.996	0.093	59.02 (56.39 to 61.64)	59.02 (56.21 to 61.97)

Shapiro–Wilk tests use normalized residuals. Sensitivity mixed-effects models retain categorical visits, final-duration adjustment, and the patient random intercept, allowing residual variance to differ by visit. Bootstrap intervals use 10,000 resamples of patient pairs. These *P* values describe residual checks, not treatment effects.

Table S7. Patient-level adverse events

Case	Event	Timing	Manifestation	Management	Revision	Outcome
1	Dural tear	Discectomy	Visible dural defect	Meticulous fascial closure	No	No wound leakage or pseudomeningocele during admission
2	Dural tear	Discectomy	Visible dural defect	Meticulous fascial closure	No	No wound leakage or pseudomeningocele during admission
3	Motor weakness	After discectomy	Ipsilateral grade-4 strength	Neurotrophic therapy; observation	No	Grade-5 strength at 2 months
4	Sensory deficit	After discectomy	Ipsilateral hypoesthesia	Neurotrophic therapy; observation	No	Normal sensation at 2 months
5	Superficial infection	Day 2	Incision redness/swelling	Dressings; antibiotics	No	Resolved; wound healed
6	Superficial infection	Day 2	Incision redness/swelling	Dressings; antibiotics	No	Resolved; wound healed
7	Screw malposition	Day 1 detection	Radicular pain; CT-confirmed canal breach	Screw repositioning on day 1	Yes	Pain resolved; no residual motor/sensory deficit
8	Screw malposition	Day 1 detection	Radicular pain; CT-confirmed canal breach	Screw repositioning on day 1	Yes	Pain resolved; no residual motor/sensory deficit
9	Anterior cortical/endplate fracture	Cage insertion	Fluoroscopic finding; asymptomatic	Bed rest; soft brace; observation	No	Healed; no migration/subsidence; Bridwell I
10	Posterior cage migration	17–24 months	Radiographic finding; asymptomatic	Observation	No	Final bone fusion; no related symptoms

Anonymous event labels are local to this table. Screw placement caused the two malpositions; symptoms, CT, and revision occurred on postoperative day 1. Neurological manifestations describe clinical severity; no retrospective formal severity grade was assigned. None of these ten patients belonged to the grade III/IV group.

Table S8. Missing assessments in the 45 excluded patients

Patient	Visit	Clinical set	LL	SL	DA	DH	FH	FA	DCSA	Bridwell
P0131	6 months	—	—	—	—	—	Yes	Yes	Yes	—
P0131	Final	—	—	—	—	—	—	—	—	Yes
P0132	12 months	Yes	Yes	Yes	Yes	Yes	Yes	Yes	Yes	—
P0132	Final	—	Yes	Yes	Yes	Yes	—	—	—	—
P0133	6 months	—	Yes	Yes	Yes	Yes	—	—	—	—
P0133	12 months	—	—	—	—	—	Yes	Yes	Yes	—
P0134	6 months	Yes	Yes	Yes	Yes	Yes	Yes	Yes	Yes	—
P0134	12 months	—	Yes	Yes	Yes	Yes	—	—	—	—
P0134	Final	—	—	—	—	—	Yes	Yes	Yes	Yes
P0135	6 months	—	—	—	—	—	Yes	Yes	Yes	—
P0135	12 months	Yes	Yes	Yes	Yes	Yes	Yes	Yes	Yes	—
P0136	Final	—	—	—	—	—	—	—	—	Yes
P0137	6 months	—	Yes	Yes	Yes	Yes	Yes	Yes	Yes	—
P0137	12 months	—	—	—	—	—	Yes	Yes	Yes	—
P0137	Final	—	Yes	Yes	Yes	Yes	—	—	—	Yes

Patient	Visit	Clinical set	LL	SL	DA	DH	FH	FA	DCSA	Bridwell
P0138	6 months	—	—	—	—	—	Yes	Yes	Yes	—
P0138	12 months	—	Yes	Yes	Yes	Yes	—	—	—	—
P0139	12 months	Yes	Yes	Yes	Yes	Yes	Yes	Yes	Yes	—
P0139	Final	—	—	—	—	—	Yes	Yes	Yes	—
P0140	6 months	—	Yes	Yes	Yes	Yes	—	—	—	—
P0140	Final	—	—	—	—	—	—	—	—	Yes
P0141	6 months	Yes	Yes	Yes	Yes	Yes	Yes	Yes	Yes	—
P0141	12 months	—	—	—	—	—	Yes	Yes	Yes	—
P0141	Final	—	Yes	Yes	Yes	Yes	—	—	—	Yes
P0142	12 months	—	Yes	Yes	Yes	Yes	—	—	—	—
P0142	Final	—	—	—	—	—	Yes	Yes	Yes	—
P0143	6 months	—	—	—	—	—	Yes	Yes	Yes	—
P0143	Final	—	—	—	—	—	—	—	—	Yes
P0144	12 months	—	Yes	Yes	Yes	Yes	Yes	Yes	Yes	—
P0144	Final	—	—	—	—	—	—	—	—	Yes
P0145	6 months	—	Yes	Yes	Yes	Yes	—	—	—	—
P0145	12 months	Yes	Yes	Yes	Yes	Yes	Yes	Yes	Yes	—
P0146	Final	—	—	—	—	—	Yes	Yes	Yes	—
P0147	6 months	—	—	—	—	—	Yes	Yes	Yes	—
P0147	12 months	—	Yes	Yes	Yes	Yes	—	—	—	—
P0147	Final	—	—	—	—	—	—	—	—	Yes
P0148	12 months	Yes	Yes	Yes	Yes	Yes	Yes	Yes	Yes	—
P0149	6 months	—	Yes	Yes	Yes	Yes	—	—	—	—
P0149	Final	—	—	—	—	—	Yes	Yes	Yes	Yes
P0150	6 months	—	—	—	—	—	Yes	Yes	Yes	—
P0150	12 months	—	—	—	—	—	Yes	Yes	Yes	—
P0151	Final	—	Yes	Yes	Yes	Yes	—	—	—	—
P0152	6 months	Yes	Yes	Yes	Yes	Yes	Yes	Yes	Yes	—
P0152	12 months	—	Yes	Yes	Yes	Yes	—	—	—	—
P0152	Final	—	—	—	—	—	—	—	—	Yes
P0153	12 months	—	—	—	—	—	Yes	Yes	Yes	—
P0153	Final	—	Yes	Yes	Yes	Yes	—	—	—	—
P0154	6 months	—	Yes	Yes	Yes	Yes	—	—	—	—
P0154	Final	—	—	—	—	—	—	—	—	Yes
P0155	12 months	Yes	Yes	Yes	Yes	Yes	Yes	Yes	Yes	—
P0155	Final	—	—	—	—	—	Yes	Yes	Yes	—
P0156	6 months	—	—	—	—	—	Yes	Yes	Yes	—
P0156	Final	—	Yes	Yes	Yes	Yes	—	—	—	Yes
P0157	Final	—	—	—	—	—	—	—	—	Yes
P0158	6 months	—	Yes	Yes	Yes	Yes	Yes	Yes	Yes	—
P0158	12 months	—	—	—	—	—	Yes	Yes	Yes	—
P0159	12 months	—	Yes	Yes	Yes	Yes	—	—	—	—
P0159	Final	—	—	—	—	—	—	—	—	Yes
P0160	6 months	—	—	—	—	—	Yes	Yes	Yes	—
P0160	12 months	—	Yes	Yes	Yes	Yes	Yes	Yes	Yes	—
P0161	Final	—	—	—	—	—	Yes	Yes	Yes	Yes
P0162	6 months	Yes	Yes	Yes	Yes	Yes	Yes	Yes	Yes	—

Patient	Visit	Clinical set	LL	SL	DA	DH	FH	FA	DCSA	Bridwell
P0162	Final	—	Yes	Yes	Yes	Yes	—	—	—	—
P0163	12 months	—	—	—	—	—	Yes	Yes	Yes	—
P0163	Final	—	—	—	—	—	—	—	—	Yes
P0164	6 months	—	Yes	Yes	Yes	Yes	—	—	—	—
P0164	12 months	—	—	—	—	—	Yes	Yes	Yes	—
P0164	Final	—	—	—	—	—	Yes	Yes	Yes	—
P0165	12 months	Yes	Yes	Yes	Yes	Yes	Yes	Yes	Yes	—
P0165	Final	—	—	—	—	—	—	—	—	Yes
P0166	6 months	—	—	—	—	—	Yes	Yes	Yes	—
P0166	Final	—	Yes	Yes	Yes	Yes	—	—	—	—
P0167	Final	—	Yes	Yes	Yes	Yes	—	—	—	—
P0168	6 months	—	Yes	Yes	Yes	Yes	—	—	—	—
P0168	12 months	Yes	Yes	Yes	Yes	Yes	Yes	Yes	Yes	—
P0168	Final	—	—	—	—	—	Yes	Yes	Yes	—
P0169	12 months	—	Yes	Yes	Yes	Yes	—	—	—	—
P0169	Final	—	—	—	—	—	—	—	—	Yes
P0170	6 months	—	Yes	Yes	Yes	Yes	Yes	Yes	Yes	—
P0170	Final	—	—	—	—	—	—	—	—	Yes
P0171	12 months	—	—	—	—	—	Yes	Yes	Yes	—
P0171	Final	—	Yes	Yes	Yes	Yes	—	—	—	Yes
P0172	6 months	—	Yes	Yes	Yes	Yes	—	—	—	—
P0172	Final	—	—	—	—	—	Yes	Yes	Yes	—
P0173	12 months	—	Yes	Yes	Yes	Yes	Yes	Yes	Yes	—
P0174	6 months	Yes	Yes	Yes	Yes	Yes	Yes	Yes	Yes	—
P0174	12 months	—	Yes	Yes	Yes	Yes	—	—	—	—
P0174	Final	—	—	—	—	—	—	—	—	Yes
P0175	6 months	—	—	—	—	—	Yes	Yes	Yes	—
P0175	12 months	—	Yes	Yes	Yes	Yes	Yes	Yes	Yes	—
P0175	Final	—	—	—	—	—	Yes	Yes	Yes	Yes

Yes denotes a missing assessment; — denotes no missingness listed for that field at that visit. Clinical set includes back/leg VAS, ODI, JOA, and SF-36. A patient may have multiple rows. Source labels 001–045 correspond to P0131–P0175. All 45 had missing imaging; 32 imaging only and 13 imaging plus clinical assessments.

Table S9. Outcome-specific missingness among excluded patients

Assessment	6 months	12 months	Final follow-up
Complete clinical set	5	8	0
LL	16	20	10
SL	16	20	10
DA	16	20	10
DH	16	20	10
FH	18	21	11
FA	18	21	11
DCSA	18	21	11
Bridwell grade	0	0	23

Assessment	6 months	12 months	Final follow-up
Any key assessment missing	26	29	36

Counts refer to the 45 data-related exclusions. Counts overlap across visits and outcomes and must not be added to obtain a patient total.

Table S10. Imaging examinations available in the analyzed cohort

Modality	Preoperative	Discharge	6 months	12 months	Final
Standing radiographs	130	130	130	130	130
MRI	130	130	130	130	130
CT	130	130	23	32	130

The denominator is 130 at each visit. Intermediate CT was performed selectively for clinical indications. FH and FA were measured on sagittal MRI and DCSA on axial MRI.

Table S11. Overall visit effects in mixed-effects models

Outcome	*F*	Numerator *df*	Denominator *df*	Adjusted *P*
Back pain VAS	907.86	4	515	< 0.001
Leg pain VAS	2326.28	4	515	< 0.001
ODI	2859.20	4	515	< 0.001
JOA	1589.76	4	515	< 0.001
SF-36 PCS	641.91	4	515	< 0.001
SF-36 MCS	308.81	4	515	< 0.001
LL	58.97	4	515	< 0.001
SL	404.94	4	515	< 0.001
DA	133.53	4	515	< 0.001
DH	730.80	4	515	< 0.001
FH	103.56	4	515	< 0.001
FA	506.92	4	515	< 0.001
DCSA	1006.17	4	515	< 0.001

Marginal Wald *F* tests for categorical visit effects. Holm adjustment covers the 13 overall tests. Each model includes 130 patients and 650 observations, with a patient-specific random intercept and final follow-up duration adjustment.
